# Nanoparticles synthesis, properties and promising devices for drug delivery systems: a review

**DOI:** 10.1186/s11671-026-04651-1

**Published:** 2026-05-20

**Authors:** Manas Barai

**Affiliations:** Department of Chemistry, School of Engineering, Dayananda Sagar University, Harohalli, Bangalore, Karnataka 562112 India

**Keywords:** Nanoparticle, Nanomedicine, Drug delivery, Drug targeting, Natural products, Tumor cell

## Abstract

This review focused on developing the nanomedicine through inclusive discussions about synthesis, physicochemical properties, in vitro and in vivo biomedical applications of different nanoparticles (NPs). Liposomal NP_S_ encapsulates the hydrophobic drugs in a lipid bilayer allowing for targeted delivery to specific tissues while reducing toxicity. The polymeric NPs are made from biodegradable material-controlled release and deliver drugs including biomacromolecules, resulting in therapeutic applications. Gold nanoparticles (AuNPs) exhibit distinctive optical and electrical properties, where they are used for targeted therapy and imaging in drug delivery systems (DDS). The current advancements focus on controlling the size, shape, and surface chemistry to develop smart NPs. Nanotechnology could provide numerous benefits, such as treating long-lasting human diseases by site-specific drug efficacy and target-oriented delivery of exact nanomedicines. Different synthesized NPs affect the lifelong activities of tumor cells, breast cancer, and several other related diseases due to their outstanding capability to control chemotherapy and immunotherapy, respectively. Drug nanomaterials are designed to deliver the target tissues resulting in declining toxicity and increasing patients’ compliance with a lower dosing index. The modern novelty of NPs lies in their ability to exhibit applications, specifically sustainable green synthesis, stimuli-responsiveness, multifunctional agents in biomedicine and environmental remediation, respectively. The integration of NPs based systems into medicine highlights their potential to develop drug delivery by improving efficacy and safety profiles in future perspectives.

## Introduction

Nowadays, nanotechnology techniques are used to improve biomedical and pharmaceutical research. First, the nanoparticles (NPs) synthesis idea was provided by scientist Richard P. Feynman in his lecture book, *“There’s Plenty of Room at the Bottom”* [1]. Nanoscale-sized compounds showed unique structural, chemical, mechanical, magnetic, electrical, and biological properties, respectively [2–7]. NPs contain three layers, i.e., surface, shell, and core, respectively [8]. Nanotechnology techniques are dependent on different classes of NPs, whose dimensions are varying from 10 to 100 nm. Different microscopic-size NPs have been shown different physicochemical properties to realize scientific attention at microscopic molecular levels. Generally, phospholipid-based NPs are strongly influenced drug-release capacity depending on different physicochemical properties, such as synergistic interaction, adsorption, macroscopic size, surface morphology, bilayer thickness and stability, respectively [9–11]. Different polymers, dendrimers, micelles, and liposomes, nanostructures can act as target-specific drugs delivery vehicles [12–15]. Due to its poor solubilization and absorption capability; drugs molecules are easily penetrated into phospholipid cell surfaces of microconidia and Goli body facilitating easy drug uptake at the target locations [16–18]. Smaller sized NPs are directly treated disease cells through decreasing the specific side effects and adsorbed on various protein surfaces then started their action on the diagnose of clinical and tumor cell [19, 20].

Recently developed nanoscience technologies are more applicable for cancer chemotherapy through DDS treatment, and imaging modalities [21–23]. Nanotechnology could offer multiple benefits in treating chronic human diseases by site-specific and target-oriented treatments [24, 25]. NPs comprising drug materials are designed at the atomic or molecular level for biochemical and cancer cell treatment. NPs are not only solved diseases of gastrointestinal region but also used to deliver drug molecules to target cells. NPs show higher oral bioavailability because they exhibit typical uptake mechanism of absorptive endocytosis. The enrichment of bioavailability is evaluated through incorporating drugs inside the target tissue, tumor, and breast cancer cells, resulting in enhance the therapeutic efficacy [26–28].

In this review, researcher has discussed that NPs are used for DDS by passing the targeted drug inside the cells [29, 30]. Various types of NPs, including metallic, polymeric, phospholipid, and dendrimer have been shown toxic effects in various tissue cells [31, 32]. Due to their unique physicochemical properties, such as nano-size distribution, large surface area to volume ratio, and functionalization ability could offer substantial important of drug delivery. The toxicity mechanism is observed due to the presence of oxidative stress, inflammatory responses, apoptosis, and dysregulation of muscle dislocation, respectively. Different sizes, surface charges, compositions, and routes of administration steps are critically influenced the cytotoxicity. Newly developed nanomedicine is necessary for understanding the toxicity profile that can ensure clinical trials in biological tissue cells. The cytotoxicity analysis is most fundamental prospective for optimizing the clinical potential and to understand the correlation between therapeutic efficacy and toxicity. For developing safer and more effective nanomedicines through studying the cytotoxicity, researchers find out different advances and interdisciplinary novelty. The green synthesis route of drug-loaded NPs is extensively stimulated, resulting in reduced hazardous constituents and side effects of chronic human diseases through site-specific and target-oriented delivery of medicines [33–35].

NPs possess significant novelty due to their unique size-dependent physical, chemical, biological, high surface area and quantum confinement effects. It is showed applications in various fields, such as medicine, electronics, materials science, drug delivery, catalysts, optical properties, controlled release, chemotherapy, immunotherapy, nanomedicine, quantum effects in semiconductors, surface plasmon resonance and pharmaceutical, respectively [36–40]. NPs are interacted with biological systems like bacteria could offer alternatives to antibiotics, antimicrobial coatings, targeted cancer therapies, etc. The iron oxide nanoparticles (FeONPs) showed antimicrobial properties used for green and eco-friendly purposes. Broad classes of therapeutics including cytotoxic agents, small interference RNA, chemosensitizer, antiangiogenic agents are carried by NPs, that effects can enhance the retention effect and permeability of exclusive pathophysiology of cancer cells. NPs accumulate in affected tissues without being recognized by glycoprotein. NPs can easily deliver chemotherapies to tumor cells at the right time, then inserted the drugs to right place with greater efficacy and reduced cytotoxicity in peripheral healthy tissues. The multitarget system has attracted extensive attention, because surface of NPs is constructed with two or more functional ligands. The synergistic interactions between NPs and cell surface could improve cell recognition and internalization.

NPs exhibit remarkable novelty due to their unique physicochemical properties and versatile applications across various fields. The gold nanoparticles (AuNPs) exhibited biocompatibility and distinctive optical characteristics making them suitable for DDS and biosensors. Similarly, magnetic NPs exhibit magnetic properties allowing for precise therapeutic interventions. Quantum dots having tunable optical and electrical properties can enhance biomedical imaging and drug delivery by enabling simultaneous targeting to affected cells. Furthermore, NP based sensors can be demonstrated high sensitivity and selectivity, which can be tailored for detecting biomolecules and environmental pollutants. Different transformative potentials including medicine, diagnostic, and environmental monitor advancing technology and health care are exhibited by NPs. NPs based DDS are designed to target specific cells or tissues through increasing the efficacy and reducing side effects. The novelty of these review lies in their ability to encapsulate drugs, protecting them from degradation and allowing for control release for cancer treatment and other diseases. Quantum dots NPs exhibit unique optical and electrical properties due to the smaller size. They showed biomedical potentials, such as cardiovascular imaging, solar cells, and light-emitting diodes fields, respectively.

## NP classes for biomedicine

NPs are classified into different categories depending on surface morphology, size, and chemical properties. NPs are produced from metal precursors, exhibit well-known localized surface plasmon resonance, and exclusive photo-optical properties [35]. Alkali and novel metals containing NPs have wide absorption band in visible region and showed high-intensity spectrum. Size and shape measurements of metal NPs are most important due to their optical natures. NPs have a higher surface area, hence easily bind with DNA and RNA of viral membrane. Inorganic NPs are used to inhibit viral growth in different pharmaceutical and biomedical research fields. The Cu is cheaper than Ag and Au, whereby AgNPs and AuNPs showed antimicrobial activity in the human body. CuNPs destroy the influenza A virus and also reduce the inactivity of feline calicivirus [41]. AgNPs are incorporated into the disinfectant cell surfaces and used as sanitizing agents in different public places for preventing virus infections. Mesoporous AgNPs can control Ag^+^ release, hence easily passed through tumor cell membrane to inhibit their cell growth and protected the virus affected kidney, liver, lung, intestine, brain, etc. The different pharmaceutical companies have been produced different biocompatible antiviral drugs, which definitely affect virus cell surfaces to control the viral diseases.

Fullerene and carbon nanotubes (CNTs) are two important carbon-based NPs [42, 43]. Fullerene is allotropic form of carbon prepared by globule hollow cage techniques. Fullerene NPs have hexagonal and pentagonal carbon units. Some other fullerenes are formed through C_60_ and C_70_ carbon units, whose diameter varies from 6.52 to 7.34 nm. The CNT's diameter was 1–2 nm and structurally resembled with a graphite sheet rolling nanotubes. CNTs formed single, double, and multiwalled carbon nanotubes, synthesized by decomposition of atomic carbon precursors using laser and electrical light by chemical vapor techniques [44, 45]. CNTs have an elongated tubular structure used for electrical conductivity, semiconductors, superficial metallic activity, gas absorption, remediation, etc.[46–48]. Different types of mesoporous silica nanoparticles (SiNPs) are obtained from various cationic surfactants, such as alkyl poly (ethylene oxide), oligomeric surfactants, quaternary ammonium surfactants, etc.[49]. The SiNPs have a rigid structure, a large surface area, and a smaller volume; those are synthesized at low temperature. The modified SiNPs obtained by PEGylation showed higher blood circulation time than unmodified SiNPs [50–52]. The SiNPs are used to deliver the drugs, DNA, and RNA to the host target cell due to their biodegradability, high stability, and low toxicity, respectively.

Iron oxide nanoparticles (IONPs) used as an efficient drug carrier to control the viral infection due to their biocompatibility and low toxicity [53–56]. The release drug from IONPs depends on pH and temperature. Release of monoclonal antibody from IONPs is faster at 5.8 pH. The IONPs could exert their biological effects through generating reactive oxygen species by oxidation, which control damage to tumor cells and decrease toxicity levels. The binding of SARS CoV-2 and hemoglobin in red blood cells is responsible for hemoglobin breakdown using IONPs. The IONPs are conjugated with mannose to form nanodrugs, that could upregulate the immune system through different activating steps as well as deliver drug molecules to dendritic cells. Super paramagnetic IONPs showed numerous applications, viz*.*, MRI contrast agents, tissue cells, immunoassays, detoxification of biological fluids, hyperthermia, drug delivery and cell separation, etc.

The non-metallic ceramic nanoparticles (CNPs) are synthesized by heating and continuously cooling processes, obtaining polycrystalline and dense forms [57]. CNPs are used as drug delivery vehicles due to their smaller size (< 50 nm), exhibiting semiconductor properties between metals and nonmetals [58]. Different varieties of semiconductor CNPs having water-splitting capability due to the presence of favorable bandgaps and band edges. They showed major applications in various research fields, viz., photocatalysis, dye degradation, nanomedicine, pharmaceuticals, biomedicine, imaging, photo optics, and water splitting purposes, respectively [59–61].

**Scheme 1:**
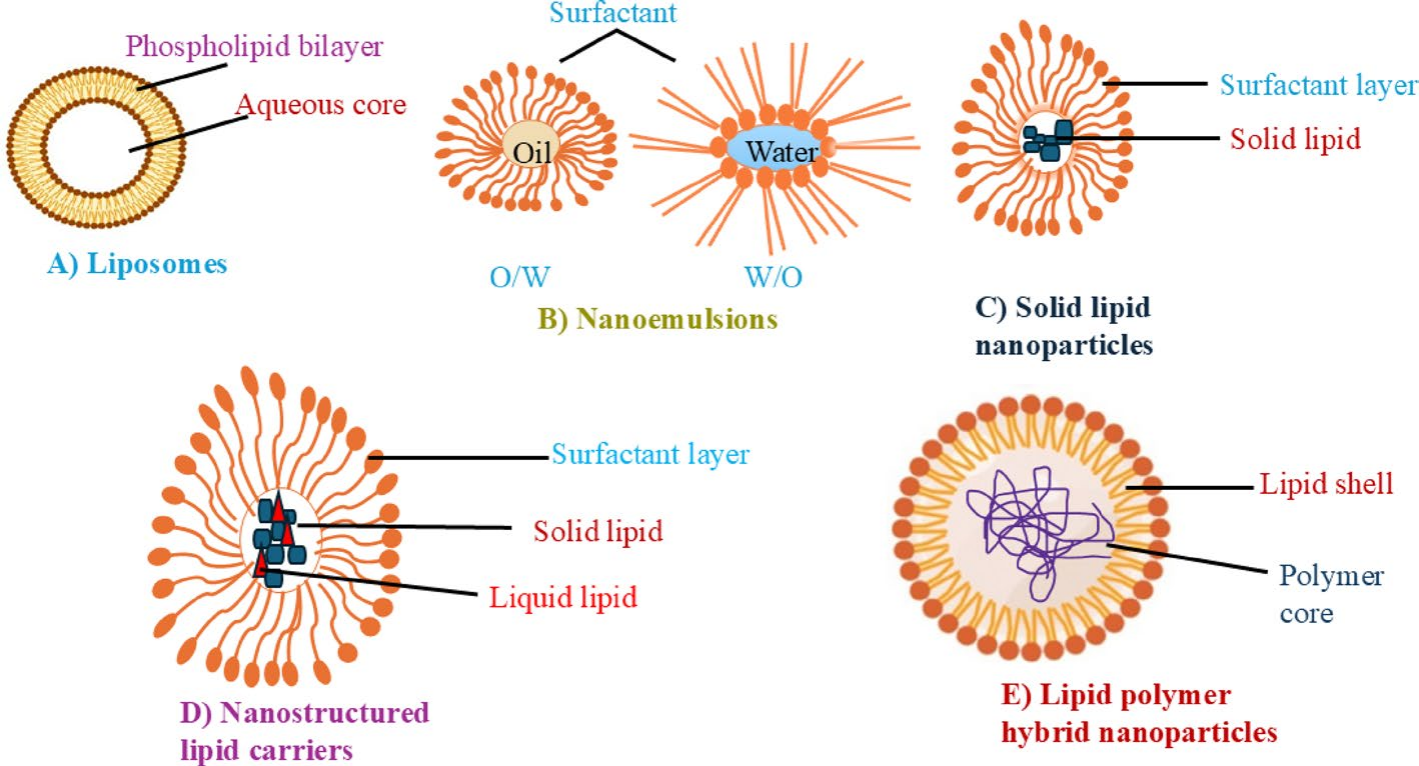
Schematic illustration depicts the structure of various LNP formulations used in drug delivery. System: **A** liposomes, **B** nano emulsions, **C** solid lipid nanoparticles (SLNs), **D** nanostructured lipid carriers (NLCs) and **E** lipid polymer hybrid nanoparticles

Lipid-based NPs (LNPs) contain soluble lipophilic polymer, whose dimension varies from 10 to 1000 nm. Nanostructure lipid carriers (NLC) can easily do their potential work in the blood circulatory system for lengthy periods as well as release drugs by controlling the plasma fluctuation (Scheme 1) [62–64].

Mono-, di, triglycerides and phospholipids are used as drug carriers; those are exhibiting hydrophobic nature in plasma membrane. The similar characteristics between the host cell membrane and the nanocarrier can minimize toxicity in the host body. A vesicle is basically used to investigate different characteristics of a nano-lipid carrier. Different phospholipids, such as phosphatidylcholine and phosphatidylserine are situated in the host plasma membrane, acting as drug carriers. The zwitterionic polar head groups of phospholipids directly attack the cell because neutral phospholipid fails to interact with cell membrane [65, 66]. The surface modification techniques of vesicles are the most functional materials for synthesizing the lipid-based nanocarrier. This modification occurs by polyethylene glycol (PEG) and stability of liposome [67]. The nanocarrier are made by mixing lipids more efficiently than solid lipids. SLNPs are used as promising agents due to presence of various shapes like cubosomes and hexosomes. Two types of cubosomes are formulated, such as (i) glyceryl monooleate and (ii) phytantriol, respectively.

Polymeric NPs are organic assemblies having nanosphere and nano capsular shapes, whose overall masses are represented in solid state [68–70]. They adsorbed photoactive light and showed different bioactive applications, whose sizes ranged from 10 to 1000 nm. They have exhibited optimistic characteristics features for an efficient drug delivery. Various synthetic polymers, such as polyvinyl alcohol, polylactic acid, polyethylene glycol, alginate, and chitosan have been demonstrated high biocompatibility and biodegradability used as drug carrier due to the hydrophilic natures. Polymeric NPs are divided into two categories, i.e., nanospheres and nano capsules used to study the biophysical and biochemical properties. Replication of the viral genome can be inhibited by conjugating the bioactive molecules with nano polymers. Acyclovir is one type of low-toxic drug molecule, associated with a polymer to reduce the viral DNA replication. Polyethyleneimine was used as an efficient drug carrier for delivering the drugs to lungs. Therefore, SiRNA conjugated with polyethyleneimine can block viral replication in influenza-infected host cells. Antigen conjugated with a polymeric nanomaterial used for vaccine development. Chitosan-coated nanocarriers with antigen showed better drug absorption capacity. Lipid polymer nanoparticles (LPNs) are another major type of lipid nanoparticle showed various biomedical applications. LPNs have polymer cores that contain therapeutic substances and lipid-PEG stealth coating for improving in vivo circulation. This specific structural administration could offer biocompatibility, physical stability, and lower toxic profiles making them suitable for an ideal DDS. The LPNs have been successfully used to encapsulate various pharmaceuticals including nucleic acid, can act as a sustained drug release through improving their stability. Furthermore, incorporating functional groups on the polymer surface is an advanced for DDS, cancer, gene therapy, vaccine development, and novel diagnostic imaging, respectively.

Many researchers have reported branch structured dendrimers containing NPs, because they are used as an efficient drug carrier for controlling different viral infections [71–75]. Dendrimers have high solubility, low toxicity and act as emerging candidates for an efficient drug vehicle. Different types of dendrimers, i.e., pegylated dendrimers, paraquets dendrimer, polyether-copolyester dendrimer, glycodendrimers are used as a nano drug carrier, synthesized by convergent or divergent processes. The divergent approach developed earlier than the convergent one. In a divergent way, synthesis of dendrimers begins from core to arm by attaching building blocks in a stepwise way. In the convergent way, synthesis started from the exterior part. The outer and inner surfaces of dendrimers have a hydrophilic nature. It binds with active pharmaceutical ingredient by hydrogen, electrostatic, and van der Waals interactions [76, 77]. Different polyesters, dendrimers composed of glycerol, natural metabolites, and succinic acid can increase biocompatibility of drugs. Cisplatin is an anticancer drug delivered to target tissue conjugated with poly(amidoamine) dendrimer showed lower toxicity and higher accumulation in tumor cells. Different biodegradable drugs, such as paclitaxel, AgNPs, and sulfamethoxazole attacks the viral receptors [78]. Organic dendrimers stimulate the host immune system through activating the cells that could inhibit the influenza virus attack in mice cells. The comparative efficacy responses (Table 1), drug formulation, target mode, comparative findings and clinical translation (Table 2) and different types of nanocarriers, their advantages and limitations, targeting strategies are briefly discussed in (Table 3), respectively.

**Table 1:** Comparative efficacy study responses by drug and theragnostic NPs

Drugs	Cellular mechanism	Subcellular localization	Mechanism of action	Cytotoxicity profile	Validation	Correlation with drug	Drug activity	Cellular uptake	Subcellular location	In vivo toxicity	References
Doxorubicin	Passive diffusion	Cytoplasm, nucleus	DNA intercalation	High cardiac suppression	MTT assay	Inhibitory concentration	Colony formation assay	Confocal microscopy	Confocal microscopy	Animal models	[79]
Cisplatin	Passive diffusion	DNA binding	DNA crosslinking	High toxicity	CCK-8	Inhibitory concentration	Apoptosis essay	Flow cytometry	Nucleus	Histopathology	[80]
Curcumin	Passive diffusion	Cytoplasm	Antioxidant	Low dose	LDH assay	Anticancer activity	Colony formation assay	Confocal microscopy	Subcellular fractionation	In vivo mice model	[81]
Taxol	Passive diffusion	Cytoskeleton	Microtubule stabilization	Peripheral neuropathy	Real time cell lysis	Intracellular concentration	Tumor growth inhibition	Flow cytometry	Localization in organelles	Body weight change	[82]
Iron Oxide NPs	Endocytosis	Mitochondria	DNA damage	High tumor toxicity	Confocal microscopy	Intracellular concentration	Apoptosis assays	Fluorescence intensity	Mito tracker	Serum biomarkers	[83]
Methotrexate NPs	Receptor endocytosis	Lysosomes	DNA damage	Reduced toxicity	Fluorescence microscopy	Intracellular concentration	Antibacterial activity	ICP-MS	Subcellular fractionation	Animal models	[84]
Ruthenium (III) NPs	Liposomal endocytosis	Nuclear compartment	Autophagic cell death	High selectivity	MTT essay	Tumor growth inhibition	Cancer growth inhibition	Optical microscopy	Localization in organelles	In vivo mice model	[85]
Liposomal Dox	Endocytosis	Nucleus	DNA damage	Reduce toxicity	Real time cell lysis	Antimicrobial activity	Tumor inhibition	Fluorescence microscopy	Mito tracker	Serum biomarkers	[86]

**Table 2:** Representation of drug formulation, target mode, comparative findings and clinical translation of different drug and NPs

Drug	Formulation	Response comparison	Nanocarrier	Target model	Comparative findings	Advantages	Clinical translation	References
Doxorubicin	PEGylated doxorubicin	Higher tumor suppression	DOX-RGD-PEG	Brain Cancer	*IC*_50_ was 0.52 mg/mL higher accumulation	NPs showed IC_50_ values lower than free drugs	Long-term effects	[79]
Salvianolic Acid	Ovarian cancer (OC)	Phospholipid NPs	PLC-NPs	Oral Cancer	Higher chemoprevention effects	Minimizing damage to healthy tissues	High production costs, low repeatability	[87]
Curcumin	Oral or beast Cancer	Lipid nanocores	Lipid Nanocarrier	OSCC Cells	Enhanced bioavailability	multidrug resistance	Multi-functional activity	[81]
Docetaxel	Cancer Cells	Nano-lipid carriers	Polymeric NPs	Breast Cancer	Drug delivery active targeting	Simultaneous diagnosis for chemotherapy	Mononuclear phagocyte system	[88]
Chlorhexidine	Cancer Cells	Nano-lipid carriers	Liposomal NPs	Cancer	Better control of infection free agents	nanoparticles concentrate drugs used tumor sites	Low repeatability	[89]
Methotrexate	Colorectal	CarrageenanAuNPs	AuNPs	Lung Cancer	Suppression of tumor growth	Encapsulated drugs can bypass efflux	Long-term effects	[84]
Camptothecin	Prostate cancer	Polymeric NPs	Lipid-based NPs	Glioblastoma	Improve stability-controlled release	simultaneous diagnosis	Long-term effects	[90]
Paclitaxel	Breast cancer	ZnO-PBA NPs	Prussian Blue NPs	Lung Cancer	92% loading efficiency	Minimizing damage to healthy tissues	High costs, low repeatability	[91]
Epirubicin	Colorectal (HCT-116)	AuNPs	Lipid NPs	Breast cancer	Enhanced bioavailability	Stimuli-responsive release in tumor	multi-functional activity	[92]
5-Fluorouracil	Prostate	Quantum dot	SLN	OSCC Cells	Drug delivery system	Enhanced uptake, targeted imaging	mononuclear phagocyte system	[93]
Metformin	Cancer cells	Nano-lipid carriers	SLN	Colon cancer	Higher biodegradability	Improved stability	Low repeatability	[94]

**Table 3:** Different types of nanocarriers, their advantages and limitations, targeting strategies are briefly discussed in the following tabular format

Nanocarrier	Typical materials	Targeting mechanism	Advantages	Limitations	References
Lipid vesicles	Phospholipids, Cholesterol	Pegylation, Antibodies, pH-sensitive lipids	High biocompatibility, biodegradable carry drugs	Poor encapsulation of hydrophobic drugs, physical instability and high production costs	[66]
Polymer NPs	PEG, Chitosan	Folic acid, peptides, aptamers, pH-responsive	High stability, control release, versatile surface modification	Potential toxicity of non-biodegradable polymers	[67]
Polymer Micelles	Au, FeO, Mesoporous Silica	Antibodies, and aptamers	Excellent for poorly soluble hydrophobic drugs, low CMC confirms stability in blood	Break apart if diluted below CMC in blood, limited drug loading capacity	[68]
Solid Lipid NPs	Lipid polymer, Inorganic-polymer	Stimuli-responsive ligand surface modification	High lipophilic drug loading capability and avoids organic solvents stability	Limited drugs loading capacity, risk of drug leakage during storage due to change the structural moiety	[64]
Dendrimers	Polylysine, PMMA	Surface-modified targeting ligands, peptides	Well-developed 3D structure, high surface functionality, size control	High production cost, and potential for toxicity	[71]
Gold NPs	Amphiphilic block and random copolymers	Target-specific ligands, pH-sensitive, tumor microenvironment	Excellent optical properties for imaging therapy, high stability	Generally poor drug loading capacity, potential long-term cytotoxicity	[95]
Magnetic NPs	Natural cell-derived vesicles	Integrins surface receptors, cell-specific modification	External magnetic field used for MRI contrast and medicinal therapy	Potential toxicity tendency requires for protective coating	[96]
Cholic acid Micelles	Amphiphilic block copolymers	Target-specific ligands, pH-sensitive, Tumor microenvironment	Hydrophobic drug delivery, Cancer therapy	High production cost, and potential for toxicity	[14]
Exosomes	Natural cell-derived vesicles	Surface receptors modification	Targeted drug delivery,	Break apart at below CMC in blood, and limited drug loading capacity	[97]

## Synthesis & surface engineering

**Scheme 2:**
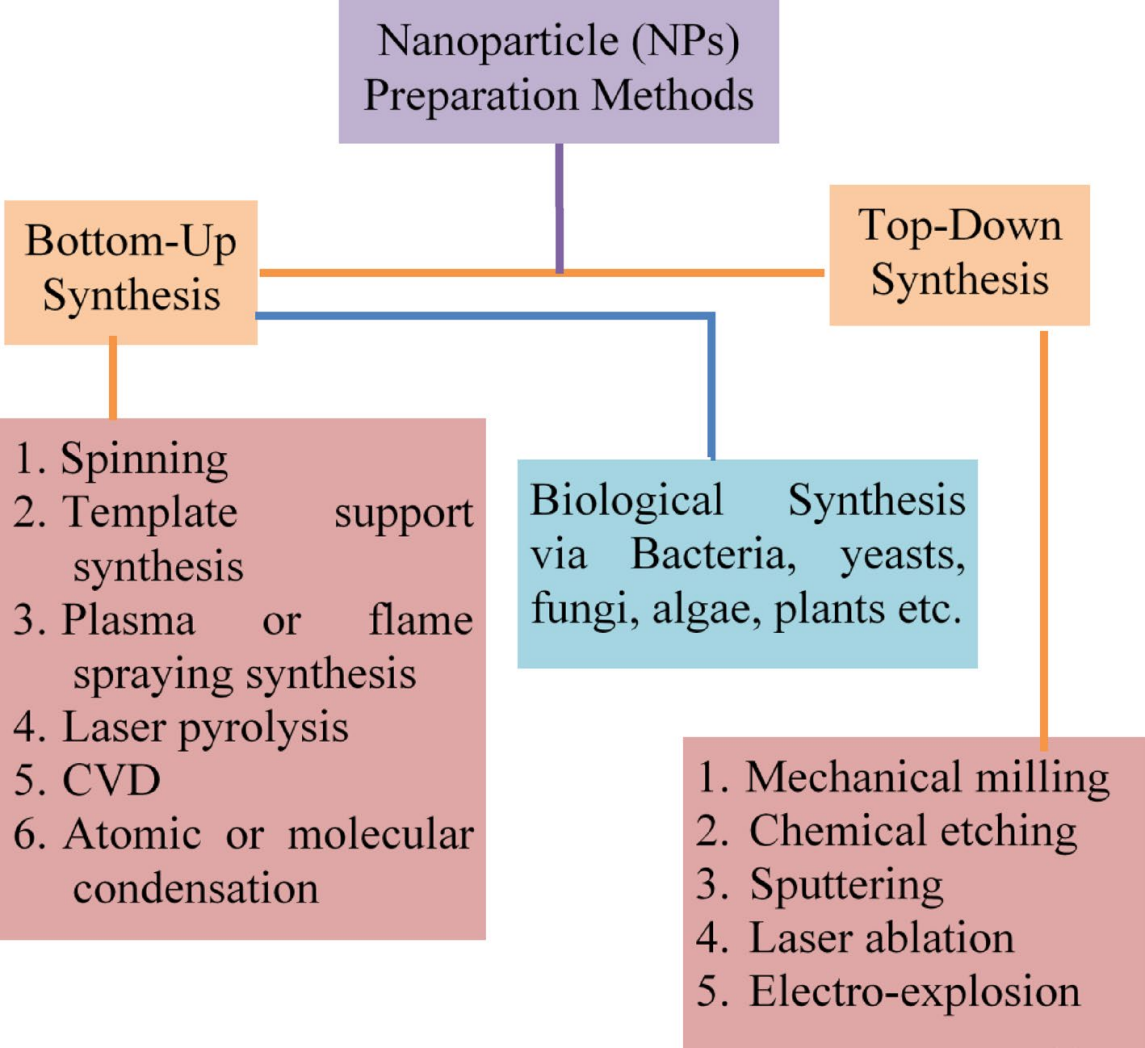
Synthetic procedures of NPs for the **a** top-down and **b** bottom-up approaches

Different synthesis methods, such as (i) top-down and (ii) bottom-up methods was chosen for NPs synthesis shown in (Scheme 2).

### Bottom-up synthesis

**Fig. 1:**
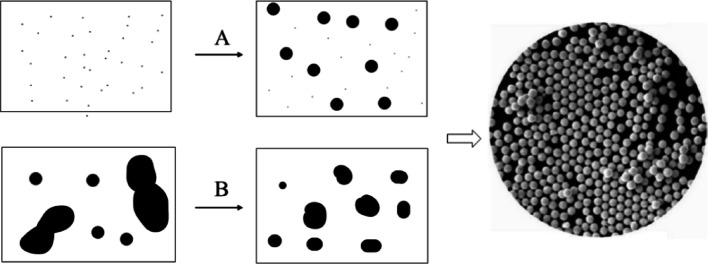
**A** Smaller molecules are fragmented to form colloids by bottom-up approach. **B** Large drops metal broken into smaller drops by top-down approach

The bottom-up approach was used for preparing the reverse NPs [98, 99]. Sedimentation and reduction techniques are best examples of sol–gel synthesis, green, spinning and biochemical product [35]. Alizarin and titanium isopropoxide used for synthesizing the photoactive composite through photocatalytic degradation of methylene blue. Alizarin was chosen to bind with TiO_2_ along with axial hydroxyl terminal groups. Spherical shapes of metal NPs are controlled by laser irradiation using top-down techniques shown in Fig. 1A. Liu et al. first selectively studied transformation of octahedral morphology through controlling laser treatment [100]. Using top-down synthesis techniques, bismuth acetate was boiled then finally produced spherical BiNPs, whose size was varied from 100 to 500 nm.

### Top-down synthesis

This method indicates larger molecules are decomposed to produce smaller units and converted to stable NPs [41, 101]. The grinding and physical vapor deposition techniques was used to synthesize the different NPs. NPs are finally milled at different time interval using ceramic and planetary ball mills, whose sizes were gradually decreased with increasing time and dimension (20–100 nm). This technique indicates frequent dispersion and formation of narrow size NPs. The powerful irradiation techniques are allowed for synthesis the well-uniform NPs (1.5–5.9 nm), shown in Fig. 1B. Adsorption processes can be produced different spherical aggregates, showing high dispersion capacity with narrow size distribution. Micrograph techniques may have reviled to the formation of smaller-size (10 to 20 nm) carbon NPs with proper sonication process. This technique indicates frequent dispersion and formation of narrow size NPs. The powerful irradiation techniques are followed synthesis of well-uniform NPs, whose size is < 5.9 nm [41]. The colloidal dispersion of AgNPs is prepared by reduction methods using different reducing agents, such as borohydride, citrate, ascorbate, and elemental hydrogen, respectively. The green synthesis of AgNPs is involved three main steps, such as (i) solvent, (ii) reducing agent, and (iii) nontoxic substances. Initially, Ag^+^ ions are reduced to AgO, then finally obtained colloidal AgNPs using various reagents [102, 103]. The emulsify AgNPs are useful substrates used to enhance surface adsorption, and antibacterial activity, because they are able to exhibit good conductivity, chemical stability, catalytic, and electrical capability. But, in presence of weak reducing agents, reduction rate of small size AgNPs was decreased.

Polymers coating NPs are controlled their surface properties, enabling enhance stability, reduced toxicity, and targeted delivery, adsorption, encapsulation, chemical grafting to tailor shell thickness, biocompatibility, and functionality, respectively. Uses amphiphilic block copolymers to encapsulate hydrophobic NPs via hydrophobic interactions can act as drug loaded nanoaggregates. Polymers are covalently attached with NP surfaces, offering greater stability than physical adsorption. The pegylated phospholipid is captures FeONPs in its core, enabling targeted drug delivery, enhance imaging contrast, and catalytic activity, respectively.

**Scheme 3:**
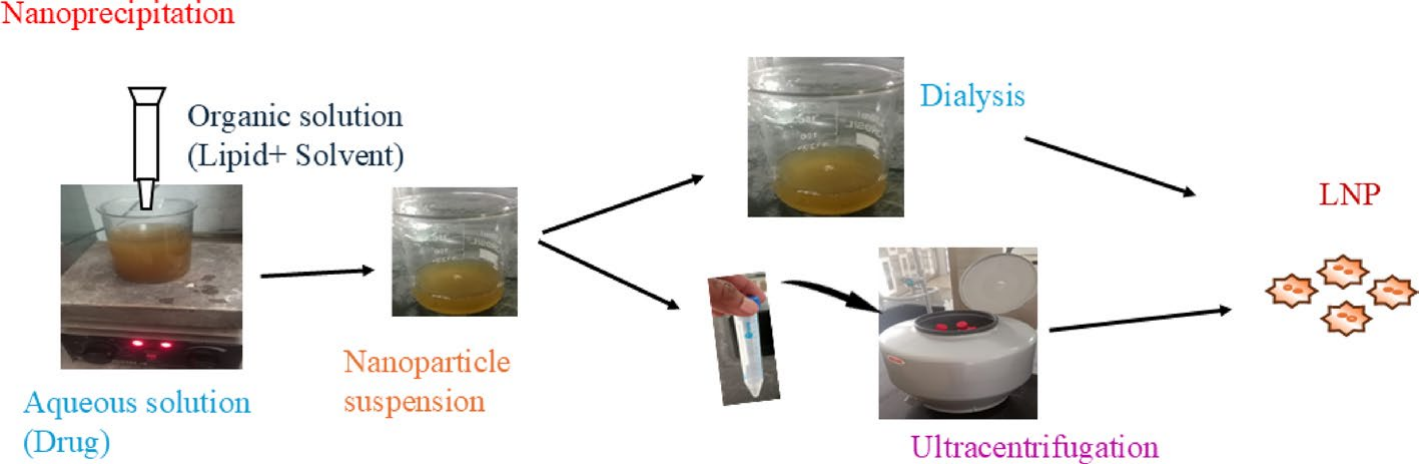
Synthesis techniques of lipid nanoparticles

Lipid based NPs (LNP) are synthesized by bottom-up and top-down approaches. For bottom-up approach used for block copolymer synthesis (Scheme 3) [104]. Using the top-down approach, bulk material is converted into nano-sized fragments using milling, spark ablation, and laser ablation processes.

The LNPs synthesis primarily followed a bottom-up approach, because they showed a distinctive colloidal structure. The main LNP synthesis methods include nanoprecipitation, emulsification, nonsolvent emulsification, thin film hydration, microfluidic process, and impingement jet mixing technology. The choice of the LNP synthesis method develops the crucial role to determine their therapeutic values by directly influencing physicochemical properties, drug loading efficiency, stability, and in vivo biological activity. Each LNP synthesis method yields NPs with exhibits distinct characteristics that directly impact their performance in various therapeutic research.

**Scheme 4:**
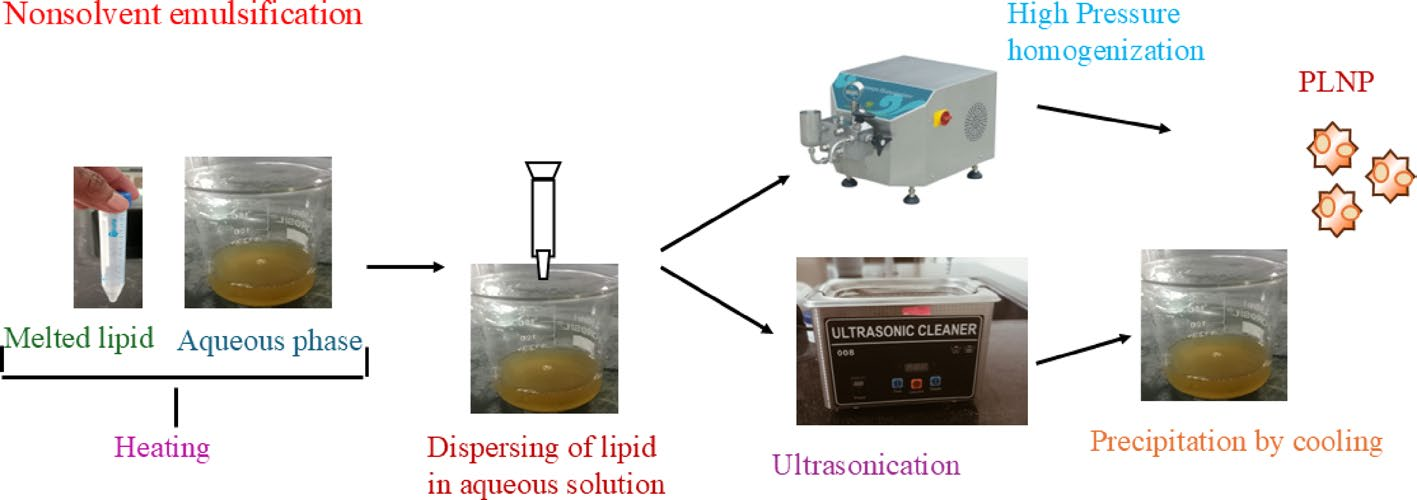
Synthesis scheme of lipid-based polymer nanoparticles

Polymeric lipid nanoparticles (PLNPs) are synthesized by a precipitation mechanism, using two miscible solvents, such as organic and aqueous phases (Scheme 4). Both are continuously mixed by magnetic stirring to form PLNPs. The organic phase consists of thin film-forming polymers, drug molecules, lipophilic surfactants, and organic solvents, but aqueous phase contains water. The size and drug encapsulation efficiency of PLNPs was prepared by nanoprecipitation through changing the various parameters, such as stirring rate, aqueous to organic phase ratio, and concentration of lipid. The PLNPs production depends on two categories, i.e., nonbiodegradable and biodegradable polylactide-coglycolide.

**Scheme 5:**
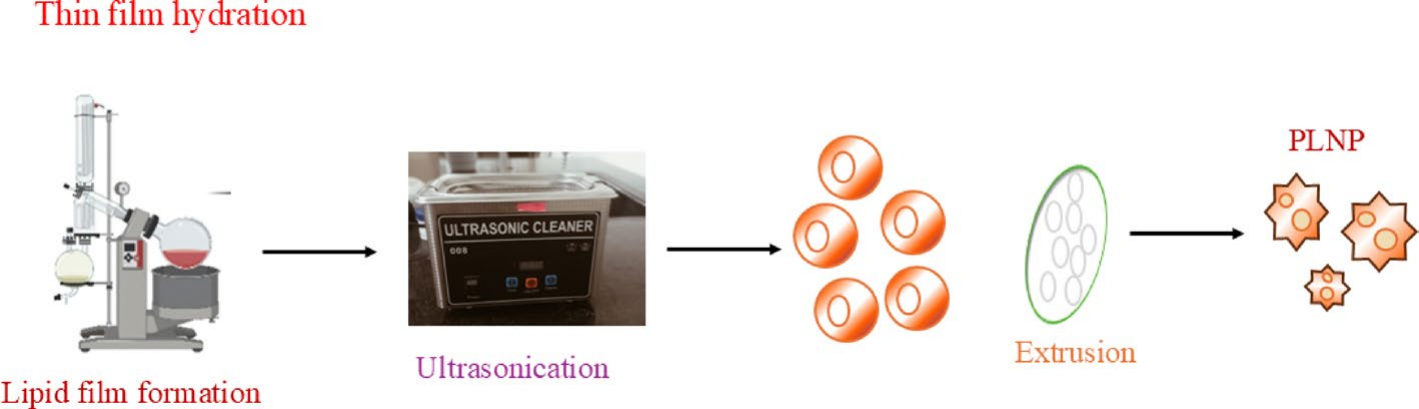
Synthesis techniques of dendrimer lipid polymer nanoparticles

After particle formation, organic solvent is removed from the formulation using dialysis bags, ultracentrifugation, rotary evaporation and freeze-drying methods. Dialysis is the most popular method for solvent removal, whereby PLNP formulation is started in a dialysis bag with a semipermeable membrane allowing solvent to diffuse in buffer solution. Ultracentrifugation techniques produce high centrifugal forces used to control size, density, leaving free lipids and incorporated drugs in the supernatant. Rotary evaporation is used to evaporate the solvent under reduced pressure and elevated temperatures. After that, the solvent was collected separately and left with the concentrated PLNP suspension. After freeze-drying, PLNP suspension was removed from the unwanted solvent through sublimation under vacuum, resulting in dry solid PLNP powder (Scheme 5) [105, 106].

These methods are crucial for PLNP formulation, ensuring highly concentrated and purified PLNPs are used for diverse biomedical applications. The formation of inhomogeneous and incompletely saturated lipid solutions may interfere with the spontaneous nucleation during small sizes of particle formation. Additionally, incomplete mixing of aqueous and organic solutions before precipitation can lead to unevenly small sizes of PLNP. By controlling crucial mixing parameters, such as speed, duration, and solvent ratio to achieve better homogenization of lipids and drug molecules. This fine-tuning ensures a more uniform distribution, resulting in consistent and efficient PLNP formulations. Henceforth, nanoprecipitation is a vital technique used to enhance the reproducibility and quality of PLNPs, making them suitable for large-scale production for various therapeutic agents.

## Physicochemical characterizations

Different synthesized NPs have been shown various physicochemical properties, such as large surface area, strong mechanical property, optical activity, and chemical reactivity, respectively. Novel metal NPs showed different photophysical properties depending on size and absorbance of light in UV–Vis region. When incident photon frequency is correlated with collective excitation of conduction electrons known as localized surface plasma resonance (LSPR). The gold nanoparticles (AuNPs) exhibit a rusty colour, while silver nanoparticles (AgNPs) are yellow, because those are freely transferable nonmaterial [95]. Magnetic properties can be identified through studying magnetic susceptibility. These techniques could measure electrical potential using homogeneous and heterogeneous catalysis, biomedicine, magnetic fluids, and data storage, respectively. Low-scale magnetic properties of NPs can effectively be used for magnetic and electrical applications. These properties are interrelated with synthetic protocols and various synthetic pathways, viz*.*, top‐down approach, co-precipitation, thermal decomposition, flame formation, and photosensitization, respectively [107]. Many mechanical properties, such as elastic modulus, tribology, surface engineering, nanofabrication, hardness are employed to study the exact mechanical nature of NPs compared with microparticles and bulk materials. Thermal conductivity of metal NPs is very much higher, displaying advance heat transfer capacity. Total surface area of metal NPs is enhanced with increasing stability. The copper oxide nanoparticles (CuONPs) and aluminium oxide nanoparticles (Al₂O₃NPs) are demonstrated better thermal conductivity in aqueous media.

### Dynamic light scattering (DLS)

DLS is a safety technique used for analyzing size, distribution, and stability of various NPs. The size is an important parameter detected accurate construction of NPs used for many biomedical applications purposes. DLS is crucial to investigate the size variation of different inorganic NPs with absorption of proteins from serum. With increasing size, the acquisition of protein layer increased due to the agglomeration and hydrophilicity, that can help to understand physical and chemical properties of NPs. DLS method used to study dynamic processes for biological and medical research and measure the size of phospholipid-based NPs used in drug delivery systems. The DLS results simplifying the analysis of large amounts of samples for study of dynamic processes. Some cases, NPs are formed aggregates, whereby their PDI values are > 5, meaning systems are heterogeneous. When they exhibit PDI < 3, system exhibits homogeneous nature and easily usable to measure the accurate size.

### Zeta potential

In disperse systems, an electrical double layer (EDL) emerges on the surface of particles at the interface. EDL is a layer of ions formed on the particle surface as a result of adsorption of ions from solution of surface compounds. The surface of NPs acquires a layer of ions of a certain sign evenly distributed over the surface with creating double charge in the NP surfaces. The potential difference between bulk fluid and stationary layer are measured by zeta potential. It can measure the effective surface charge and predicting colloidal stability. Zeta potential values above − 30 mV typically indicate good stability due to electrostatic repulsion, while values between − 10 mV to + 10 mV suggest the aggregation. Zeta potential is highly sensitive at higher pH, ionic strength, and concentration. It’s can measure electrophoretic mobility of particles in an applied electric field to understanding negative surface charges in environmental and biological systems.

### Transmission electron microscopy (TEM)

**Fig. 2:**
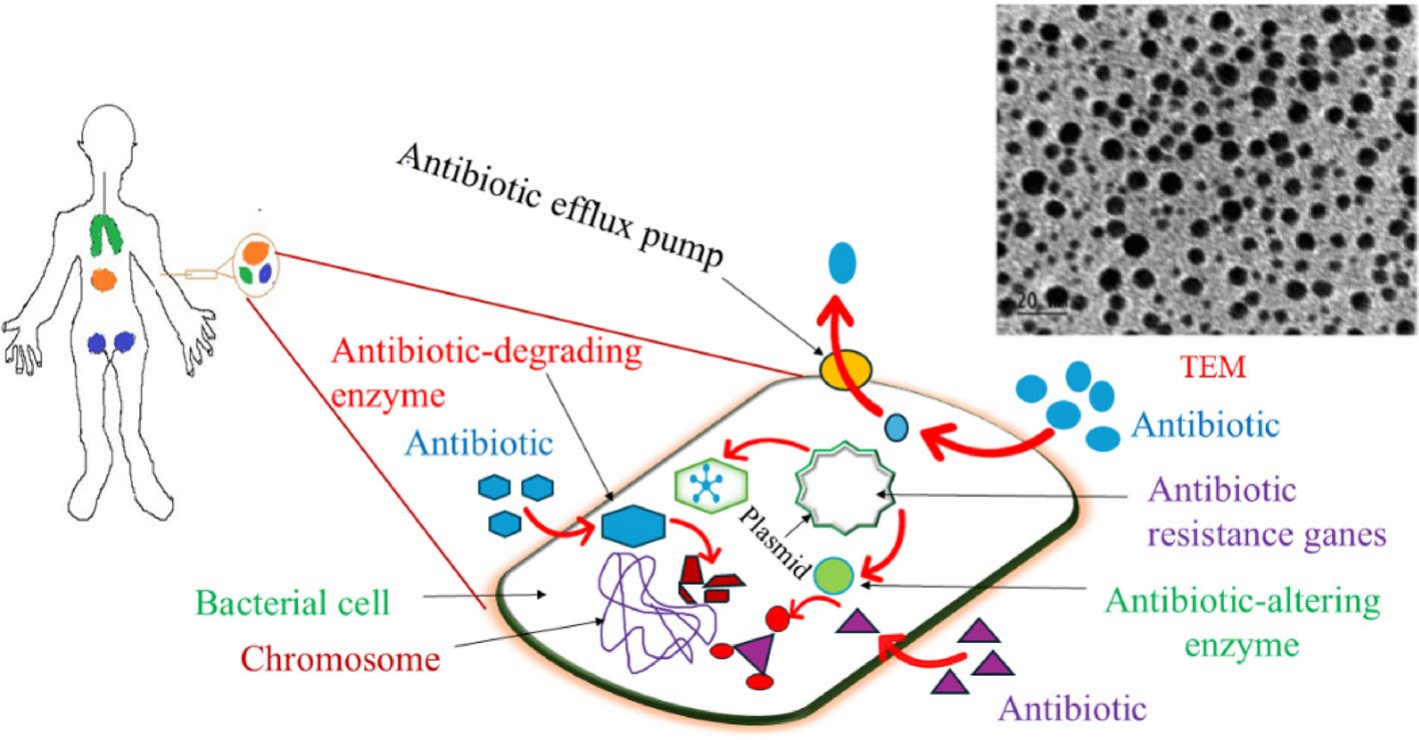
Expected body distribution of intravenously administration of particles sizes confirmed by TEM analysis. Systems: Olive > 7 µm; orange = (0.1–7) µm; and blue < 6 nm, respectively

At first dilute, pure dispersed suspension of nanocomposite mixed with polar protic solvents and applying a small drop onto a carbon-coated copper grid. The grid is dried to form a thin layer using for imaging. At first, sonicate the NPs suspension (0.1–1 mg/mL) for several minutes to break their aggregates form, then 5–10 µL of suspension is put onto the grid, often resting on filter paper to absorb excess liquid. Allow the sample to dry in vacuum desiccator to remove solvents and reduce contamination. Different size nano drug aggregates are mostly used to control the diseases in different organs in body. In here, Fig. 2 showed TEM images of AgNPs; synthesized using negatively charged heparin as a stabilizing agent by heating at 70 °C for ∼8 h. TEM images of AgNPs revealed that particle size was gradually increased with increasing AgNO_3_ and heparin concentration. Furthermore, changes in heparin concentration, size and morphology were varied significantly, where heparin used as a nucleation controller and stabilizer. After characterizing the size, NPs are absorbed into the tissue cells and organs through proper channels used for DDS. Preferably, NPs have reached the target anatomical site; after that, the immune system contributes by the recognition of reticulum endocytosis [108]. Alternatively, mechanical filtration by lungs, liver, and kidneys can lead to activities of lipid-based NPs. Henceforth, their size measurement is very important for in vivo animal model tests. A schematic representation of particle distribution after intravenous administration is shown in Fig. 2.

When NPs sizes are larger than 7 μm, those are easily filtered and entrapped in lung surfaces. But, when particle sizes are varying in the range of 0.1–7 μm, showed reticuloendothelial system is liver or blood vessel [109]. If, the particle diameter is lower than 100 nm, it may remain constant in blood vessels, since the possibility of NPs is reduced on molecular cell surfaces. The smaller sized NPs with a diameter of 6 nm can easily pass into the kidney and started drug-releasing activities through glomerular filtration methods. But, smaller-sized AuNPs are better used for in vivo cell viability and cellular internalization due to the presence of different in vivo characteristics [66].

### Physicochemical characteristics to expected biological effects

**Scheme 6:**
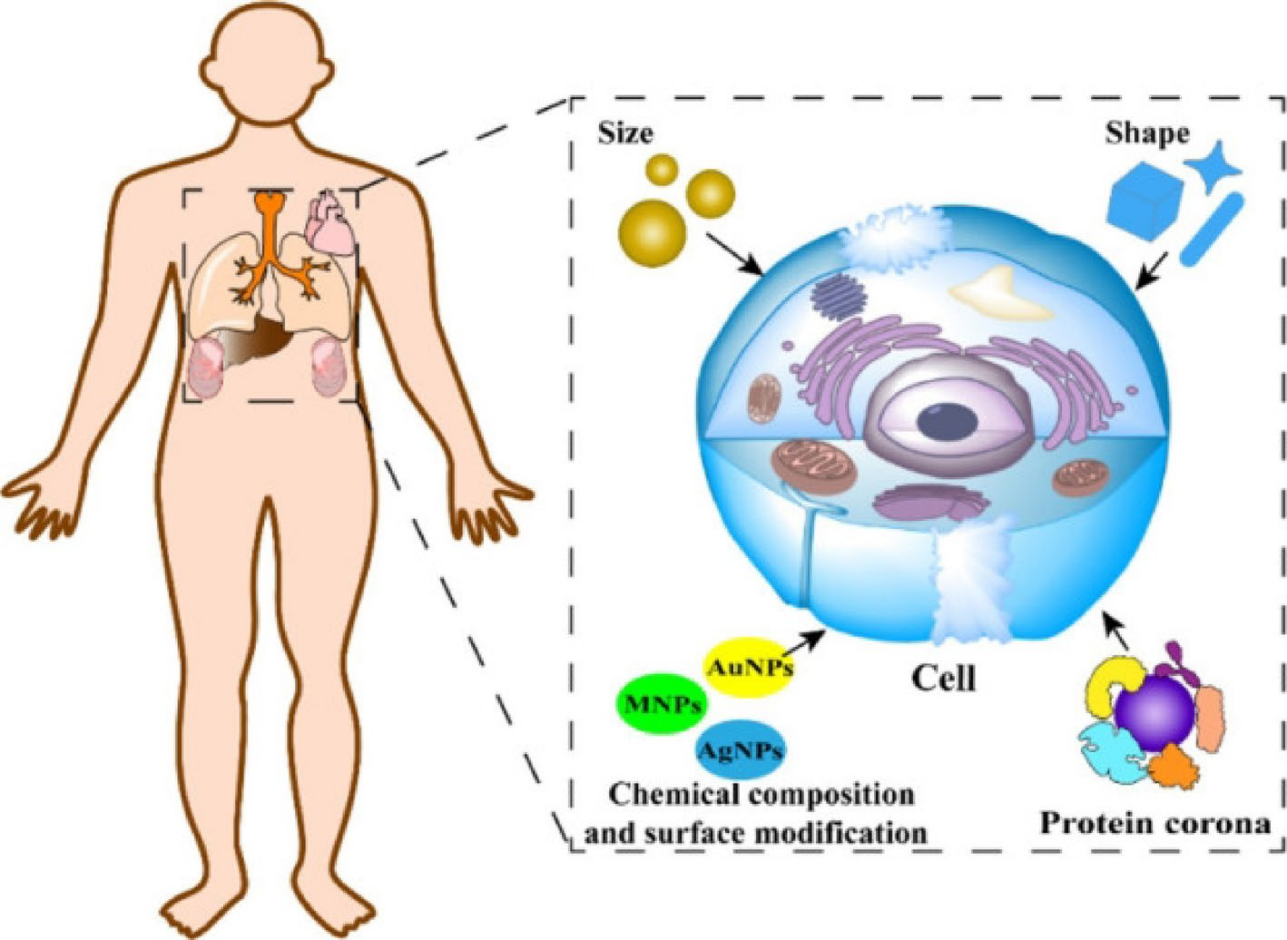
Model-based mechanistic framework described physicochemical characteristics is corelated with biological effects of various NPs [82]

Physicochemical characteristics are foundation parameters used to predict surrogate drug behavior within biological systems by influencing pharmacokinetics. The molecular weight, particle size, shape, chemical composition, and surface properties of NPs control the extent of drug absorptions into the bloodstream to estimate mammalian toxicological kinetics. Key phytochemical descriptions have served as novel surrogates to predict drug response, absorption, membrane permeability, and distribution, respectively. A model-based mechanistic framework of phytochemical characteristics to the biological effects is shown in (Scheme 6).

Physicochemical properties govern cellular uptake pathways, protein corona formation, and formation of reactive oxygen species (ROS) to determine ultimate biological effects. NPs enter the body, then interact with plasma proteins, tumor cell receptors, and small biological molecules to start the therapeutic action.

#### Size

Different size NPs affect the release kinetics, half-life in blood, uptake by immune cells, metabolism in kidney, leakage from reticuloendothelial system, etc. [110, 111]. The size of NPs could measure the specific surface area whereby they enter into the tissue cells through phagocytosis and pinocytosis mechanisms. Larger sized NPs ~ 500 nm are specially participated via phagocytosis. On the other hand, smaller sized NPs are taken more in cell membranes via pinocytosis, showing higher toxicity because they have a larger surface area that can enhance the catalytic activity in between cell membrane and NPs. Different size of NPs controlled intracellular processes for normal cell growth and homeostasis pathways that control the molecular damage, oxidative stress, etc. The non-cellular factors, like different size can control cellular reactions and interaction between NPs and mitochondria respiration, immune response, and ROS-mediated kidney damage, respectively. AuNPs with varying the size in between 20 to 35 nm are usually used as an antibacterial agent affecting toxicity. Zapór et al. [112] assessed the cytotoxic of AgNPs with varying different particle sizes, such as 10, 40, and 100 nm. They observed that smaller size AgNPs caused mitochondrial dysfunction and increased cell membrane permeability due to the development of oxidative stress. The AuNPs have a high electron density, dielectric properties, catalytic effect, and ability to bind with various biological micro molecules without affecting their activity and phytochemical properties. Therefore, size of NPs are strongly influences different attractive performance and application to directly affect the surface curvature and signaling pathway of cells.

#### Shape

NPs showed different shapes, such as manosphere, nanorod, nanowire, nanosheet, nanotube, nano cube, etc. When NPs enter inside the organisms, their shape can influence the molecular dynamics and in vivo toxicity via different phagocytosis steps [113]. Lu et al. [114] found that when NPs were passing through the pulmonary surfactant layers are affected by NPs, due to the presence of spherical shape. However, the shape of NPs plays major roles in translocation and pulmonary perturbations. With varying the shape of NPs, efficiency of translocation decreased. The cuboid tetrahedral NPs have sharp corner shape, those are penetrated the pulmonary surfactant layer with a slight structural modification. NPs of other shapes exhibits lower efficiency of transportation. Synthesized TiO_2_ NPs exhibit different shapes used to study the biodistribution, accumulation, and toxicity when intravenously administered to healthy mice [115]. Additionally, bipyramidal and plate-shaped NPs displayed higher accumulation, but rod-shaped NPs leads histopathological pulmonary alterations. Additionally, they have increased the serum biomarkers associated with hepatocellular injury. The smaller shape NPs substantially alter their accumulation and safety efficacy in the tissue cells.

#### Chemical composition

At various compositions, NPs inserted into the tissue cells, microorganisms influence toxicity via different phagocytosis pathways. When NPs were passing through the pulmonary surfactant, they started therapeutic efficacy in a patient’s affected cancer and tumor cell surfaces. NPs play a fundamental role in translocation and pulmonary surfactant perturbations into the affected tissue cells. AuNPs with varying compositions could exhibit lower efficiency of transportation, as characterized by the distance between NPs and the pulmonary surfactant layer [113]. Synthesized AuNPs exhibit different sizes with varying compositions, whereby used to study the biodistribution, accumulation, and toxicity when intravenously administered to healthy mice in vivo [95].

#### Surface modification

The surface modifications can also cause different biological effects. Dias et al. [113] synthesized AuNPs coated with citric acid (CA) and tannin acid (TA) to compare the effects of surface ligands on blood adsorption. The surface charge data of both types of AuNPs exposed to serum proteins showed significant protein adsorption. The AuNPs interacted with TA formed small clusters and induced low uptake. On the other hand, AuNPs combined with CA produced smaller granular by endosomal mechanism that indicates surface ligands of AuNPs have unique biological interactions [116]. The researchers established surface chemistry of ultra-fast superparamagnetic FeONPs significantly affected protein adsorption, endothelial cell uptake, viability, and barrier function, respectively. Although carbon nanotubes showed different surface extensively used in medicine and biotechnology for several decades. Lu et al. [114] investigated the potential side effects and changed the surface modifications of pristine multiwalled carbon nanotube (P-MWNTs) and oxidized multiwalled carbon nanotube (O-MWNTs) to assess specific macrophages to exhibit significant cytotoxicity, reduced cell viability, and promoted apoptosis, respectively.

### Standardization of NPs

Based on nanotechnology techniques standardization of NPs is well-known process ensuring the reproducibility, safety, and compatibility across various laboratories and industries. To standardize the size, shape, surface charge, purity, drug loading and release rates are critical to develop laboratory research for clinical advance therapy practices [117]. Standardization is crucial to confirm safety, efficacy, reproducibility, quality control and important surrogate markers for drug response. The standardized NPs may establish surrogate endpoints with associating clinical, dermatology, drug releasing and efficacy properties. NPs based diagnostics can enhance the detection of disease biomarkers to improve the accuracy and sensitivity for diagnostic tests [118]. Additionally, integration of standardized NPs into personalized medicine allows for tailored treatments based on individual patient body by enhanced the therapeutic efficacy. The reproducibility of nanoscience technology is particularly established for clinical translation studies to address variability in synthesis and enable commercial scale-up. The international organization has standardized the NPs with dimensions in between 1 and 100 nm. Different standard techniques measured the physical properties, i.e., DLS for size, SEM and TEM for surface imaging, respectively. Development of certified reference materials is crucial for calibrating instruments to ensure consistency across different research industry. Standard operating procedures are necessary, particularly in green or plant- synthesis to ensures consistent and high-quality production by standard source materials. Standardization of NPs could provide guidelines on how NPs are interacts with biological environments for instance, regulatory compliance, therapeutic efficacy and DDS [119].

### Reproducibility and quality control

The reproducibility process is most challenging for preparation of highly sensitive NPs [120, 121]. The small-sized NPs can alter the drug-loaded micelles to the specific targeted sides to enhance the therapeutic efficacy. Regulatory bodies strongly recommend larger size NPs production, where manufacturing processes meet quality targets rather than final product. Microfluidic technologies are being adopted to improve size distribution and reproducibility of large-scale production. Reproducibility and quality control measurements are vital to clarify the reliability of novel surrogate markers for drug response in biological systems. This process generates the reliable data for the development of a surrogate marker for drug responses across different laboratories. The integration of standardized practices and rigorous validation processes can enhance reliability of surrogate markers through improving drug development. The reproducibility process highlights the significant challenges due to biological context with varying drug and growth conditions. Identifying and controlling these factors is essential for improving reproducibility in cell-based assays, implementing good laboratory practices and using precise analytical methods for accurate measurement of surrogate markers. This approach provides a competitive advantage by ensuring meaningful data for drug development. Standardization protocol is necessary to achieve high intramolecular DDS and enable successful replication. The comparison between reproducibility and quality control is crucial to identify the genomic predictors.

### Regulatory considerations

The study of regulatory considerations is focused on the multidisciplinary field confirming human and environmental safety. The key considerations involve standardization, characterization, assessing unique toxicological profiles, and developing adaptive frameworks in nanotechnology fields. Regulatory considerations for NPs are varied by jurisdiction, product type, categorized as either medicinal product. Regulates nanoproducts under existing frameworks like the new drug application (NDA) process are assumed to detect cytotoxicity. European medicine agency (EMA) assesses medicines on a case-by-case basis through discussing with a specialized multidisciplinary expert group. Another European chemical agency (ECA) manages the safety of manufactured nanomaterials under regulation acts to include nonspecific requirements. The pharmaceuticals and medical devices agency (PMDA) produces the qualitative nanoproducts by collaborating internationally followed the essential nanotechnology guidance. International standard organizations (ISO) provide critical guidelines for standardized testing and terminology for phytochemical characterization. Regulators require finding out detailed ideas about the size, shape, surface chemistry, and aggregation state, whereby DLS and TEM are essential for these mechanistic techniques. Studies on regulatory considerations could provide ideas of how NPs are absorbed, distributed, metabolized, and excreted, specifically looking for accumulation in liver or brain. Regulatory consideration frameworks for nanodrugs are evolving to address their complexity focusing on safety and efficiency [122]. The regulatory considerations are generally applicable to tissue cell development rather than single molecules, due to the diverse effects of nonmedicinal. Regulatory consideration must emphasize the drug requirements as well as show unique biological aspects, such as specific biodistribution, interactions with the immune system and potential for accumulation in tissue cells [123]. Regulatory considerations concerning surrogate markers are essential in drug development, particularly when traditional clinical parts are impractical in tissue cells. Surrogate markers are biological indicators controlled the DDS and facilitated early decision-making and regulatory approvals for life-threatening conditions. Surrogate markers can expedite the approval process for drugs targeting the cancer cell lines, reduce blood pressure, and cardiovascular diseases. Different regulatory body parts are needed for laborious validation of surrogate markers to confirm the clinical profits.

### Drug discovery and design

Drug discovery and design are amended issues for developing nanotechnology research. Different therapeutic methods are projected to increase drug specificity for treating cancer and tumor diseases through attacking the specific target regions. The reducing toxicity and increasing bioavailability in organism are an essential topic to provide accurate information about nanotechnology [124]. Based on nanoscience technology, proteins, peptides, and biological targeted nanoclusters can act as nano-based medicine used for therapeutic efficacy with controlling the death-like diseases. Several studies and reviews have been focused on rational drug design of different molecules to exhibit the importance of drug release mechanism. Natural products have been achieved interesting solution properties for addressing drug design and discovery through changing physicochemical properties [86]. Interestingly, drug delivery systems have their own chemical, physical and morphological characteristic, shown affinity of drug potential through chemical interactions, covalent bonds, hydrogen bonds, electrostatic and van der Waals interactions. Meanwhile, drug delivery systems have gained significance novelty to release active ingredients from the body. Chen et al. [125] described imaging and sensory applications of NPs to exhibit therapeutic effects for the treatment of different diseases. Additionally, Pelaz et al. [126] discussed therapy effect of nanomedicine and reflected new prospects of these systems. Mattos et al. [127] elaborated the drug release profile of biogenic SiNPs grafted with neem bark extract-loaded biogenic SiNPs. Sethi et al. [128] demonstrated cross-linkable lipid shell containing docetaxel prototypical drugs used for controlling the discharge kinetics in both in vivo and in vitro conditions. Kamaly et al. [36] discussed control drug release from polymeric nanocarriers. Apart from this, different composition of nanocarriers is formed drugs showed drug delivery profile against gram-positive bacteria. Hence, all these factors can influence the interaction of nanocarriers with biological systems, and release kinetics of active ingredient in the organism. Several studies regarding drug release mechanisms have connected with diffusion, solvent, chemical reaction, and stimuli-controlled release, respectively. Although there are several nanocarriers with different drug release profiles can improve the mobility of NLC for targeting the organism with phospholipids. Salatin et al. [129] highlighted the endocytosis mechanism responsible for the cellular uptake of polysaccharide NPs. It has been noted that NPs are used as a drug carrier using different paths, such as phagocytic, non-phagocytic, clathrin-mediated endocytosis, caveolae-mediated endocytosis, etc.

### Drug loading

**Scheme 7:**
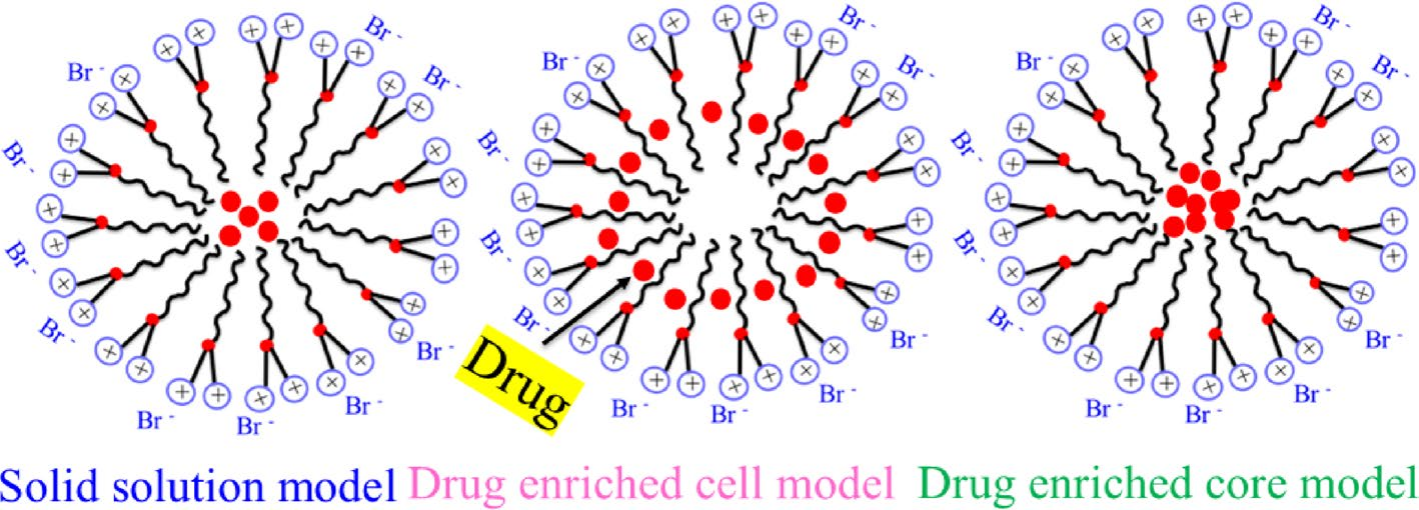
Drug loading caping agents

The drug loading mechanisms of different nanocarriers are analyzed by frequent administration routes. But, the oral route cannot be used to deliver drugs, because they may degrade different digestive tracts. The drug delivery are performed using different caping agents, such as phospholipid, dendrimer and polymer aggregates exhibited low solubility, increased bioavailability and decreased cytotoxicity, respectively (Scheme 7).

The drug molecules easily passes inside the cell surfaces, then released into the appropriate places of effective organs through controlling the activities [64, 108]. The recent emergence of innovative strategies of nano drug carriers can control several diseases. For example, lignan is a synthetic stem cell used to treat rheumatoid arthritis and multiple sclerosis viruses. An antiviral polymeric nano micelle-based drugs are conjugated with specific ligands to produce single-chain polymers with coated on the upper surfaces of virus, resulting in putting to death the influenza, HIV, and other viruses [124, 130]. In most cases, chitosan is chosen as a drug carrier shown muco-adhesive properties used to control the diseases in epithelial junctions [131]. Chitosan is biopolymer which exhibit individual drug loading properties in presence of biocompatible functional groups. It is easily encapsulation various types of NPs for detection and diagnosis of diseases. Thus, chitosan-based nanomaterials are broadly used buccal, intestinal, nasal, eye, and pulmonary, respectively [132–134]. Alginate is another biopolymeric material derived from brown seaweed used as a drug delivery carrier, exhibited different physicochemical characteristics, such as low-cost manufacture, pleasant nature, lower harmfulness, and easy responses, respectively. Due to the presence of carboxyl units, these biopolymers have represented greater mucoadhesive strength [135–137]. Patil et al. [138] develop insulin-containing alginate NPs with nicotinamide as a permeation agent. The alginate nanogels used for contrast-enhancing agents and expected to be suitable drug carriers in pharmacology. On an urgent basis, different severe diseases are obtained by virus attacks in different organs through changing DNA and RNA translocation; henceforth, we can explore the drug release by therapeutic pathways. But DNA and RNA have shown a rapid mutating nature and biologically diverse characters. To overcome these problems, carbon quantum dots are used as a promising agent, as they have unique potent antiviral activity by tuning several heteroatom doping. The antiviral NPs are spherical shapes and may exhibit greater antiviral effects. The mercaptothion sulfonate functionalized bovine serum albumin coated tellurium NPs used to control the side effects of porcine epidemic diarrhea virus [139].

Drug release and distribution kinetics are obtained through studying the absorption of drugs for therapeutic efficacy and pharmacokinetics [140]. The overall process involves by drug liberation steps through releasing the dose from blood vessels, followed by absorption into the bloodstream, and subsequent distribution to body tissues. The rate of drug release is a critical parameter that can determine different therapeutic efficacy through controlling the diffusion, erosion, and swelling matrix, respectively. Many control drug release systems exhibit an initial burst through rapid release of surface-bound drug by slower and sustained release. Oral administration route confirmed how many fractions of drugs reached the tissue cells by systemic circulation. Firstly, oral drugs are passing in metabolism of gut and liver. Only an unbound drug can move from blood to tissue, that can act as therapeutic agents used for infected tissue cell development purposes. Protein-bound drugs can act as a reservoir used to control the central compartment blood model, two peripheral compartments by well-occupied tissue cell interactions [141]. The control drug release formulation can easily maintain stable plasma concentrations through minimizing cytotoxicity. The control drug release formulations are desirable easily release the drug in the lower track region, resulting in reduce the total amount of absorption compared to interstation release. Firstly, the control drug release formulations are sometimes passed into the metabolism by releasing the drug in the colon, after that passed through the tissues and central plasma compartment while maintaining the longer drug effect.

#### Drug distribution

Drug distribution kinetics describe how nanomedicine is liberated and moves through body compartments [142]. Different key factors, including matrix swelling, erosion, blood flow, protein binding, and carrier structure are influenced the distribution processes. According to the Ficks law, drugs diffuse through a polymer matrix can exhibit porous structure through diffusion-control processes. The drug distribution kinetics rate was analyzed using zero-order constant rate, first-order, Higuchi and Korsmeyer-Peppas models. Using these different models, higher surface area to volume ratios of NPs allows rapid initial release from specific target-oriented sites for long-term delivery. Drugs bound to plasma proteins like albumin cannot easily distribute while free drugs readily move into tissues [143]. The drug distribution techniques are controlled through changing age, body composition, and vascular permeability diseases significantly, where lipophilic drugs distribute more readily into tissues containing high fat.

#### Drug metabolism with time

**Fig. 3:**
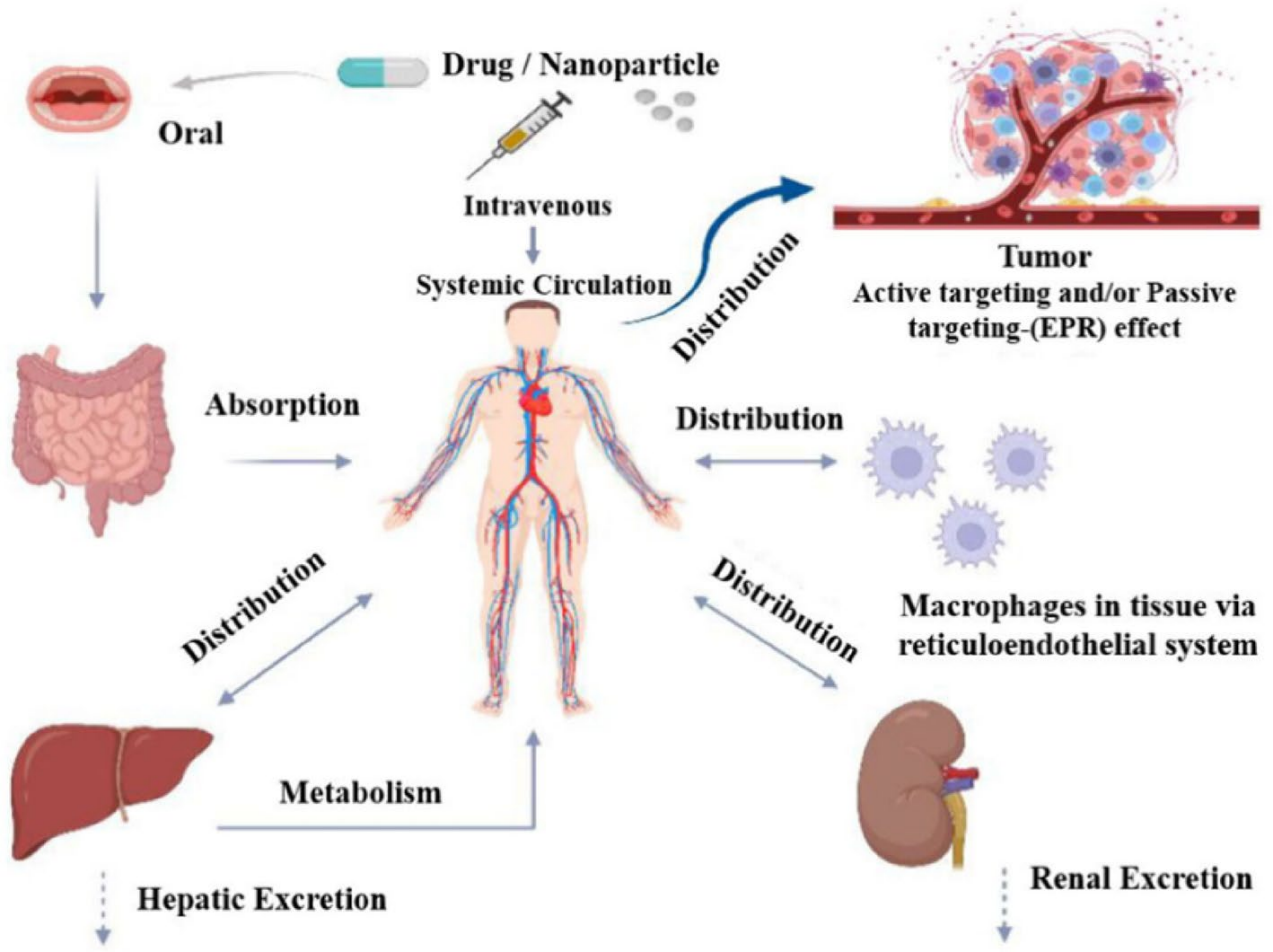
Drug load, distribution, metabolism, and release of NPs [140]

Drug metabolism with time is chemical alteration route of drug enzymes in liver. Those are removed by oxidation, reduction, or hydrolysis using cytochrome P450 enzymes. Adding a polar moiety, such as glucuronidation or sulfation making them suitable for more water-soluble for excretion [144]. On the other hand, prodrug is an inactive compound that is metabolized into an active form through enhancing the metabolism in tissue cells. Other drugs like substances, such as grapefruit juice and smoking can speed up or slow down metabolism through interactions. The oral drugs are metabolized by reducing bioavailability in liver (Fig. 3).

Different byproducts are obtained from several body parts through time-dependent drug metabolism techniques, showed active, inactive, and toxic properties, respectively [145, 146]. The metabolites are biochemically active showed therapeutic effects, but inactive metabolites have not shown therapeutic and toxic effects due to their inactivity. Toxic metabolites are biochemically active compounds shown various harmful effects. Drug metabolism occurs at a specific location in the body resulting in lower concentration of active metabolites used for systemic blood circulation [147]. This phenomenon happens through passing the metabolism in target sites showing limited bioavailability. Firstly, metabolism is primarily passed through the liver to body. The kidney is primarily responsible for excretion of drugs from the body; however, lipophilic drugs readily cross the cell membrane by absorbing the blood molecules. Therefore, lipophilic drugs are first metabolized in liver before excretion drug. The metabolism of drugs occurs by various reactions and electrostatic repulsion between the drug molecules and targate molecules. Those are categorized by modification, conjugation, addition and excretion techniques. Using modification techniques, the chemical structure of lipophilic drug is altered by different physicochemical properties through oxidation, reduction, hydrolysis, and cyclization through removing the polar molecules [148]. In this process, an inactive prodrug changes its biological effect metabolically, where the active drug is complied with other bioactive molecules.

Drug metabolism over time can act as a surrogate marker of efficacy and safety drug response. It is determined the pharmacokinetic profile of drug in biological systems. Time-dependent enzyme metabolism induction or inhibition can directly influence the drug releasining rates through therapeutic adversements. Baseline metabolite profiles can predict a patients likely response, that effecte can help to select appropriate dosages or alternative therapies. Time-dependent inhibition of enzymes can predict potential drug-drug interactions and drug-induced liver injury, which are common reasons for clinical trial failure. Knowledge of metabolic pathways helps to identify soft spots molecule, guiding structural modifications to optimize pharmacokinetic properties and reduce toxicity. The rate of formation and elimination of metabolite is a better predictor of pharmacological effect than the parent drug alone. The rate of formation of reactive or toxic metabolites serves as a surrogate marker for potential safety liabilities. Repeated dosing can induce enzymes, leading to increase clearance, reduced drug exposure, and potential therapeutic.

#### Drug release

The drug release patterns are operated by zero and first-order kinetic rate equations. Drug elimination is an irreversible technique, helped to remove the drug from our body comprising with metabolism by chemical modification and excretion techniques [149]. Metabolism primarily occurs in the liver. The stimuli-responsive nanocarriers have been shown controlled drug release ability using ultrasound, heat, magnetism, light, pH, and ionic strength, respectively [150–152]. Different inorganic NPs are connected with polymeric nano lipids to stimulate the control drug release. Ulbrich et al. [153] reviewed drug delivery mechanism of polymeric and magnetic NPs and addressed the effect of covalently or noncovalently attached drugs for cancer treatment [154]. Therefore, different hybrid nanocarriers can use for nanomedicine through enhancing the therapeutic and diagnostic activities in tissue cells. The synthesis of environmentally safe nanocarriers from plant extracts and microorganisms real mechanism of drug delivery can open up the newer avenue in pharmaceutical research. The drug molecules are targeted to a particular location of therapeutic agents to overcome the surface barriers by mononuclear phagocytosis. The NLC is embattled at a specific site in the body. The significant amount of drug is released because they are captured in a hydrophobic environment by passive and self-delivery pathways. Meanwhile, drug release profiles are directly conjugated to carrier nanostructured material. In these cases, drug release is a crucial process because it will not reach the target site and quickly dissociate from the carrier. Henceforth, its bioactivity and efficacy gradually decreased and released from tissue cells. The main target receptors of cell membranes are lipid components, antigens and proteins on cell surfaces. Currently, most nanotechnology-mediated drug delivery systems are targeted for cancer, diagnosis, detection, and imaging purposes, respectively. The integration of therapy and diagnosis is extensively utilized for cancer treatment. In addition, NPs are used as therapeutic agents for tumor cells, which can provide necessary action about molecular stimulus response.

The drug release therapy showed promising treatment, that can offer different benefits, such as a lower dose of an individual drug showing few side effects, accomplishing numerous complementary therapeutic goals, and lowering risk of resistance [65]. Nanocarriers are also naturally helpful for the distribution of various medications with different physicochemical properties. The drugs-loaded nanocarriers have been designed with superior control due to their flexible capability, overlapping pharmacokinetics, and decreasing adverse effects. Drug release is an important property of a therapeutic system. Firstly, the drug is absorbed in a targeted cell, then contributes to the therapeutic availability. The oxidation, reduction, and hydrolysis steps of drug involve cytochrome P_450_ enzymes and Phase II conjugation reactions in water. Excretion mostly occurs in the kidneys via glomerular filtration, active tubular secretion, and reabsorption, respectively. The main novelty of these systems is to maintain the concentration of therapeutic agents in the blood or in target tissues as long as possible. Therefore, the control drug release system can initially be a fraction of the dose that can attain the effective therapeutic concentration of drug release. The drug release kinetics can maintain exact mass transport mechanism that can predict quantitative drug release kinetics to design pharmaceutical formulations by designing the new systems. It is clear that the development of a mathematical model requires the comprehension discussion of drug release kinetics. Generally, when a hydrophilic drug is incorporated in a matrix, drug release occurs easily by diffusion compared to another hydrophobic water-soluble drug. The solubility of drugs can explain their active nature, where a polymeric matrix is incorporated showing different biological behaviours. Considering the qualitative and quantitative changes that may alter drug release and in vivo performance. In this regard, the use of in vitro drug dissolution can predict in vivo bio performance by considering the rational development of control drug release.

## Bio-nano interactions

### Protein corona

The Dawson research group first studied the interaction between NPs and plasma proteins. The NPs are replaced by protein corona (PC) then exhibited electrostatic interactions with hydrophobic drug molecules [155, 156]. This PC is primarily composed protein can acts as biodegradible substances in the presence of different biomolecules, such as sugar, nucleic acid and lipid, respectively. Initially, NPs are interacted with biological fluids before reaching the plasma then started actions with tissues to expose the active biomolecules through transforming bare NP into a biological PC [157]. At first, PC molecules are absorbed protein, after that, biomolecules are accomulated around the lung surfaces, then enter the bloodstream. The NPs fluids are passed inside the gastrointestinal tract in presence of enzymes that not only hinder the bioavailability but also demonstrates continuous NP-PC complex formation. The blood plasma is established with proteins in the plasma membrane. The PC is devided into hard and soft corona based on time change mechanisms of compoundes. The protein corona profile find out the differences between pancreatic cancer patients and healthy individuals. The hard corona is strogly interaced with proteins at long exchange time, but soft corona is showed second layer of proteins an over time. The composition of hard corona, particularly the ratio of opsonins to dysopsonins serves as surrogate marker an immune system. By characterizing the hard corona, researchers are studied the predict biodistribution, cellular uptake, and therapeutic efficacy, respectively [156]. Protein adsorption and surface natures of NPs with PC can act as a crucial surrogate marker of drug responses because they establish the biological identity through interacting with cells and tissues. When NPs enter biological fluids are immediately coated with proteins, lipids, and other biomolecules to form new biological nanomedicine to enhence the ability and efficacy of tissue cells [158]. The NPs based therapeutics have been used in new area in translational medicine. Despite the clinical success, it is not well to understood how NPs fundamentally change the biological environments in presence of physiological fluids [159]. Those are potentially developed new NPs through altering their bioactivity, stability, and destination properties. Additionally, the conformation of protein is highly sensitive due to the interaction with NPs, that can change the conformation and orientation of proteins. The corona typically contains specific proteins, which can bind to the upper surface of NPs like albumin or apolipoproteins. PC formation is not static, where high-concentration corona adsorbed protein is initially absorbed then coated with NPs surfaces. But due to the presence of highly surface-active substances, corona is gradually replaced by lower-concentrated, higher-affinity protein. The time-dependent dynamics of protein corona occurs through adsorbing the soft corona proteins and free proteins in the surrounding medium. With changing the pH, temperature, and other biological mediums, corona protein compositions could exhibits biodistribution, cellular uptake, toxicity, etc. [160].

The composition and dynamics of PC layers are adsorbed onto NPs surface upon contact with biological fluids to determine the in vivo darmatological activity, biodistribution, cellular uptake, respectively. The efficacy and toxicity of DDS confirmed that the protein corona is interacted with cell membranes by reticuloendothelial system. The specific proteins adsorbtion can predict the efficacy of systemic therapies to distinguish between cancer stages. The corona is not static proteins initially adsorbed then gradually replaced by higher-affinity proteins over time to understand how NPs are targeting after administration. The protein corona is influenced by individual patient factors to serve as a personalized, patient-specific marker for developing more precise and effective DDS [161]. The PC encouraged the absorption of NPs in tissue cells and led to the pro-inflammatory response of macrophages. The novel targeting method of drug delivery confirms the specific delivery of NPs within the bloodstream to determine the undesirable effects on the formation of protein corona. Initially, corona can alleviate the interactions with serum proteins, and after that, removal of the NPs from the bloodstream. The NPs are used in different drug delivery research when they are shielded with a protein corona layer to attack the target specific sites. The formation of a protein corona can not only change the biological characteristics and targeting of NPs but also change the properties of proteins. Zao et al. [162] showed that human serum albumin (HAS) was the main binding protein in blood, and AgNPs could adsorb HAS to form stable corona protein on AgNPs surfaces. Using spectroscopic techniques, researchers showed that the secondary structure of protein are greatly improved in the DDS. Investigating the effect of NPs on protein conformation is not only help to reduce toxicity of NPs, but also reveal the mechanism of DDS [163].

### Stability in biological fluid

NPs stability in biological fluid is most prominent aspect for analysing the functionality, biodegradability and higher stability, respectively [164, 165]. Polymeric NPs can break down the electrostatic interactions between fluid and nanocomposite, resulting in trigger instability, enhance chemical properties through interacting with serum proteins. Different bioactive and low-toxic lipid, sugar, nucleic acid and protein are interacted with NPs to enhance stability, because they are formed PCs. Here, researchers addressed the most common strategies of biological fluids to visualize NPs dispersion, high ionic strength and antifouling strategies, respectively [166, 167]. Stability in biological fluids can act as surrogate markers of drug response in biological systems particularly to develop the pharmaceutical research. The integration of biological markers into therapeutic drug might be enhanced the precision therapy. Stability in biological fluids is a critical factor in drug delivery, ensuring that therapeutic agents remain active, protected from premature degradation, and capable of reaching their target site within the human body. Drugs, particularly mRNA, proteins, viral vectors are degraded rapidly in blood by enzymatic cleavage, hydrolysis, and oxidation, respectively. NPs encapsulate drug can act as a physical shield against degrading enzymes. Those are coated with polyethylene glycol creates a hydration layer that prevents the plasma proteins binding to drug, reducing macrophage uptake and increasing circulation time, respectively. When a drug is intravenously administered in gastrointestinal fluids the combat pH fluctuations ensured the drug releases. Surfactants and antioxidants are added to formulations to prevent the drug from breaking down due to ROS or water contact. A stable DDS must balance long circulation time to release drug molecules at the target site. The stability of the NPs depends on its ability without prematurely releasing the drug. Stability in biological fluids refers to drugs ability to maintain its structural integrity, chemical potency, and physical form while traveling through the human body complex internal environments. In drug delivery can ensures the medication reaches its intended target without being prematurely degradation. Biological fluids showed several challenges for drug stability through several mechanisms. Many advanced therapies like mRNA or proteins are highly unstable and destroyed by enzymes before starting the work. Carriers like LNPs encapsulate drug act as physical shield against these external threats. Blood consists of serum proteins are attached with liposomes through opsonization and eliminates liposomes from the system. Additionally, it interacts with various plasma components and enzymes of blood due to immune system response [168].

### Drug half-life

The concentration of drug in plasma membrane is reduced half of the initial level after the half-life time. The relationship between the percentage of drug elimination and the number of half-life is also important for developing the drug delivery in tissue cells through improving the therapeutic efficacy. The concept of half-life realised on several key assumptions including drug metabolism, hepatic deficiencies, drug-drug interactions and alternative metabolic pathways, respectively. In addition, patient age is a significant factor for determining the accurate half-life of drug, especially for pediatric patients. On the other hand, drug metabolism and half-life can vary significantly for healthy middle-aged adults to determine the drug dosing frequency and maximum incubation in tissue cells [169, 170]. The half-life of drug studies is very essential for determination the excretion rates and steady-state concentrations of active bioactive species. A longer half-life means the drug stay inside the body for a longer time, exhibiting less frequent doses, but shorter half-life needs more regular maintenance of effectiveness and enhancement [171, 172]. The liver, kidney and other medication can influence the half-life of drug molecules through changing the molecular interactions. After an initial dose no interactions happened between drugs molecules, because they can control health problems in certain quantitative by first-order pharmacokinetics. The clinical decision is very challenging task in nanotechnology research due to the highly theoretical nature. Researchers have realized that half-life calculation is more effective and safty for studying the pharmacological modality of tissue cell diseases through controlling therapeutic efficacy [173]. Drugs with a short biological half-life needs frequent dosing to achieve long time therapeutic response. The main goal is to maintain the therapeutic blood level over extended periods for entering the drugs easily into tissue cells. Therefore, half-life is used to estimate how long it take drug to remove the diseases from our body. To understand the drug development by half-life is most vital for designing effective therapeutic efficacy. In here researcher focused the DDS studies through studing the optimal half-lifes, that can help to improve the efficacy, safety, and patient convenience, respectively. For sustained drug release are enhenced by extend the half-life of drug, that can enhence the therapeutic effect and reducing the frequency of dose. Similarly, short time drug formulations used to achieve rapid therapeutic effects too avoid oscillations in drug levels. In cases of renal failure, drug excretion is enhenced leading to increase peak initial concentration and excretion rate of drug molecules. Hepatic disease also affects the half-life of drug due to impaired metabolism. Half-life is also clinically relevant when clinicians must establish the most effective and safest dose to achieve an optimal therapeutic effect.

### Cellular uptake kinetics

The cellular uptake kinetics could exhibit the extracellular transport of NPs at a constant concentration [174, 175]. In most cases, the kinetic rate equation is not able to describe the bioavailability because diffusion process cannot alter the rate equation and mostly related to the square root of time. The transport of NPs in an extracellular medium is divided by three steps, such as diffusion, sedimentation and fluid flow inside the bulk medium. Molecular diffusion of NPs is observed when interacted with other extracellular medium to exhibit completely random motion in preferable direction [176]. If, NPs exhibit lighter dose capability in surrounding medium, particles may be moved upwards direction other than sedimentation. The actual sedimentation rate also depends on NPs size, making these processes more important for extracellular transport by controlling cellular uptake kinetic rate [96, 177]. Cellular uptake kinetics function exhibits critical predictive and surrogate markers of drug response based on quickly and extensively enters the drugs inside the cell. The cytotoxic effects can identify mechanisms of drug resistance and optimize drug delivery systems, particularly for targeted nanotherapeutics. The total amount of drug has taken by cells over time to determine the cell death rate using only short-term, early-stage, or low-concentration uptake data. In drug-resistant cell lines increased cellular uptake rates, often achieved through targeted delivery systems can directly correlate with enhanced cytotoxicity. Because most drug targets are intracellular, knowing the rate of entry is more informative for predicting response than measuring extracellular concentrations. Cellular uptake kinetics are used to evaluate NP surface modifications, maximize internalization and therapeutic efficacy.

### Blood–brain barrier

The NPs are specialized for drug delivery systems designed to pass into the blood–brain barrier (BBB) [178, 179]. NPs utilize several physiological pathways used to enter the brain through receptor-mediated transcytosis. The NPs are functionalized with different photoactive ligands, such as transferrin, insulin, respectively. They easily bind to specific receptors on endothelial cells, triggering internalization and transport across the barrier. In the presence of highly selective semipermeable membrane, NPs are naturally excluded over 98% of small-molecule drugs. Polymer, lipid-based NPs, vesicles, SLN, and mimic biological membranes are highly stable and biodegradable for delivering mRNA and other nucleic acids. They have easily crossed BBB then released the drug molecules. Studies on stability and surface modification techniques of AuNPs could improve imaging and deliver chemotherapeutics to specific brain regions using external magnetic fields. Fluorescent active NPs are used for real-time imaging for brain tumor and usable for biological processes. Adsorptive-mediated transcytosis showed electrostatic interactions between positively charged cationic NPs and negatively charged luminal surfaces of BBB. Charge carrier-mediated system can transport proteins for nutrients like glucose or amino acid linked NPs into the brain. Using physical disruption techniques, microbubbles are passed through the tight junctions of BBB via non-invasively. In recent years, nanotechnology research has been helpful to solve the diseases of major central nervous systems to deliver targeted bevacizumab and doxorubicin like chemotherapy inside the affected tissue cells. Recently developed BBB crossing lipid NPs are capable of delivering the functional mRNA to neurons [180]. The BBB plays a critical role as a predictive marker of drug response in biological systems particularly context of neurological disorders. Its permeability directly influences the efficacy of DDS to central nervous system for developing effective treatments. Furthermore, disruption of BBB can enhance drug delivery, serving as another predictive marker for therapeutic response. Meanwhile, cerebrospinal fluid and neuroinflammation biomarkers could provide valuable insights into drug mechanisms and anti-inflammatory effects. Techniques like positron emission tomography imaging further complement these assessments by visualizing drug distribution and target engagement in the brain is enhancing the drug efficacy. The importance of BBB is assessed by biomarkers through predicting and evaluating drug responses in neurological contexts.

### Dose response biological effects

The dose–response biological effects of NPs are mostly correlated with cell death, characterized by non-linear dynamics, where surface area of surrogate dose is directly determined from biological effects [181, 182]. The internalized dose can exhibit accurate correlation with membrane. Cell death typically follows a non-linear model, where lower doses may trigger hormesis, while higher doses exceed a specific biological threshold level through irreversible apoptosis pathways. On the other hand, smaller size NPs with higher reactive surface area could exhibit stronger dose dependent toxicity. As for example, AgNPs exhibit apoptosis at lower doses (< 50 μg/mL), but necrosis happens at higher concentration (> 80 μg/mL). NPs are used as novel surrogates followed by trigger specific regulated cell death pathways including ferroptosis, cuproptosis, and pyroptosis, respectively. Through delivering the metal ions by inducing ROS is dose-dependent manner. They easily induce immunogenic cells by transforming cold tumors into hot bodies by releasing damage associated molecular patterns through stimulating immune system. The dose response relationship is complicated in nanotechnology, where NPs bioprocessing by ion dissolution in blood vessels alters the surrogate inside the biological system [183]. The dose–response relationship is complicated to determine the continuous physicochemical transformation of NPs induced biological system. Whereby, to solve this problems, present review addressed the relationship between dose responsive elucidate properties and discusses how dynamic transformation breaks the NPs at subcellular levels. The dose response biological effects can act as predictive and surrogate markers to understand the drug responses by physiological processes. These are measurable biological responses used as substitutes to find out the direct clinical survival morbidity. They are expected to predict clinical benefit based on established scientific evidence. Biomarkers derived from dose–response curves guide the selection of optimal therapeutic doses to balance efficacy and safety. Biological effects confirm that drug is targeted the molecules to solve the serum creatinine kidney injury as well as act as a safety biomarker to detect potential toxicity before damage.

Lethal doses of ultrafine FeONPs depend on material size, whose size was less than < 5 nm [184]. Lethal doses of dietary AgNPs caused developmental delay and profound lethality in animals and young adults. In contrast, exposure to sublethal doses does not develop animal cells, shortening adult lifespan and compromise tolerance to oxidative stress. Importantly, AgNPs are accumulated in tissue cells, after that ROS is activated by an in vivo antioxidant pathway, that can control the variety of ROS mediated stress response apoptosis, DNA damage, and autophagy, respectively. The lethal and sublethal doses of AgNPs have been shown acute and chronic effects on development long performance capability by inducing ROS mediated stress. The molecular and cellular mechanism of AgNPs showed adverse effects at the organismal level to explore toxic effects and mechanism of organismal, cellular and molecular levels using drosophila model system [185]. In recent years, NPs have achieved significant effects and showed lower doses than traditional free-drug counterparts due to enhance cellular uptake and targeted delivery [186]. Half maxima inhibitory concentration is the dose required to inhibit a specific biological function. For instance, nitric oxide-releasing SiNPs could exhibit intravenous invasion, where targeted FeONPs have been achieved tumor cell invasion by unbound inhibitors. Unlike traditional drug loaded NPs have biphasic responses, where low doses might stimulate cell growth while higher doses cause inhibition. Conjugating NPs with antibodies can increase localized concentrations at target sites, which are required for systemic dose purposes. Using NPs formulations, researchers have successfully used lower doses of inhibitors compared to oral administration through maintaining tumor growth inhibition and significantly reducing metabolic effects [187].

Drug response biological effects control drug delivery in presence of safe and effective administration parameters for development of stimuli-responsive systems. They have released the therapeutic agents in response to specific physiology. Different biological factors, including pH, enzyme activity, temperature, and redox conditions are allowed for proceeded the DDS. They minimize the premature release of drugs to control diseases in tissue cells. Premature release of drugs bound to the surface of a nanocarrier can occur, which could result in negative effects on other tissues. Conversely, drug release from the DDS matrix is contingent upon biodegradation kinetics. The release mechanism of drug response biological effects are three phases, such as (i) burst release of drugs near the surface; (ii) little release after adsorption on tissue surfaces and (iii) successive burst release due to the particle disintegration. Traditional DDS, such as tablets, capsules, and syrups, are characterized by rapid elimination from the body, resulting in erratic drug levels within the therapeutic range. These techniques confirmed that the drug release happens by swift metabolism, leading to an immediate increase in drug levels.

### Comparative IC_50_ values

**Fig. 4:**
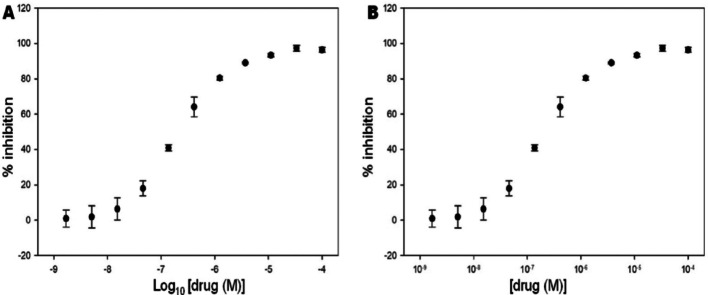
**A** The dose response curves plotted with log10 [drug (M)] and **B** molar drug concentrations for inhibition of bovine alkaline phosphatase [188]

The dose response IC_50_ figure showed important insights into potency along the dose axis. That can yield important parameters such as half-maximal inhibitory concentration (IC_50_). The IC_50_ is the concentration of inhibitor required to show 50% inhibition. IC_50_ is highly dependent on the assay conditions and unable to be interpreted in molecular frameworks. Apart from the measurement of concentration of the chemical species, small-molecule drug metabolite and efficacy are important mechanistic obtained from drug release concentration (DRC). The slope of transition can shed important insights into the mechanism of action how the small molecule interacts with the target protein. The dose response curves plotted with log10 [drug (M)] for inhibition of bovine alkaline phosphatase schematically shown in (Fig. 4). In contrast, the log10 value of drug depends on molar concentration of drugs. produce IC_50_. The DRC data has profound implications for both how data are estimated approximate distribution and corelationships between different compounds.

The IC_50_ values control drug delivery by acting as standard for potency, supervising the design of dosage, and optimizing release kinetics in targeted delivery systems. It defines the concentration required to inhibit different biological processes, such as cell proliferation and enzyme activity to determine the precise amount of drug needed to exhibit the therapeutic effect. Dosage optimization and potency is the primary sources for measuring the drug potency. A lower value indicates that a drug is more potent to produce the desired effect. These studies directly informed that doses allow for reduced systemic toxicity while maintaining efficacy. Targeted delivery system design in advanced drug delivery is used to calculate the efficiency of drug-loaded NPs compared to free drugs. Sustained release kinetics is a known drug. Engineers can design delivery systems that maintain the drug concentration for a prolonged period, improving therapeutic outcomes, particularly in anticancer and antimicrobial treatments. Evaluation of efficacy in different environments can monitor real-time cell adhesion and detachment after drug administration. It is also used to compare drug efficacy between two-dimensional cultures and three-dimensional tumor models, providing a more accurate estimation of dosage required for in vivo applications. The controlled release rate is tuned to ensure the concentration in the target organ to optimize the pharmacological response.

### Therapeutic efficacy

Therapeutic efficacy controls drug delivery is controlling the release rates to maximize therapeutic effect while minimizing toxicity. Modern DDS use smart materials, such as nanocarriers to enhance solubility, protect from degradation, and target diseases in tissue based on pH or enzymatic triggers, rather than relying on conventional dosage forms. Formulations like polymers and matrix systems release drugs at a predetermined rate to maintain constant therapeutic levels in bloodstream, preventing of toxicity and ineffectiveness in conventional dosing. Advanced delivery uses ligands or antibodies are functionalized with nanocarriers, such as liposomes and dendrimers to deliver medication directly to disease sites, such as cancer cells, reducing systemic side effects and improving safety, respectively. By modifying how a drug is distributed, metabolized, and excreted, these systems improve bioavailability, allowing for lower and less frequent dosing, which increases patient compliance. Therapeutic efficacy aims to enhance the pharmacokinetics, target tissues, and drug release profiles. Novel DDS reduce dosing frequency, improving convenience, improve patient adherence and better treatment outcomes. Targeted DDS showed better advantages while minimizing systemic side effects. They can selectively deliver drugs to tumor sites, inflamed tissues, or specific cells within the body, resulting in improved therapeutic outcomes and reduced toxicity. This techniques enhance the drug concentration in the body, reducing the frequency of dosing and maintaining therapeutic levels over a longer duration.

Several drugs showed potential therapeutic efficacy with considerable toxicity and little efficacy. As a result, nanotechnology techniques are used to achieve the boost in therapeutic activity by drug-loaded medicine and reducing toxicity. Therapeutic molecules are changing construction of different usable nanocarriers making them suitable for drug candidates. The production of prodrugs assured compatibility, inclusion into specific nanocarriers and controlled release techniques. A nanocrystal-formulated antiviral prodrug, and cabotegravir shows remarkable effects in pharmacokinetics and prolonged drug release in vivo mice model. Kulkarni et al. [65] reported that emtricitabine is another highly water-soluble prodrug used for in vitro to in vivo extrapolation modeling due to its continuous release in targeted cells. Therapeutic efficacy devices can directly input the nanosized drugs into the body, providing sustained and localized drug release over an extended period, resulting in reduced dosing frequency, improve patient convenience, and enhance compliance, respectively. Due to the achievement of drug delivery capability through discussing with therapeutic efficacy, researchers have been focused on developing patient-friendly formulations, such as oral control release systems, transdermal patches, and inhalation devices. These formulations aim to simplify drug administration, improve convenience, and enhance patient adherence to prescribed treatment in the future perspectives.

### Targeted cells

**Scheme 8:**
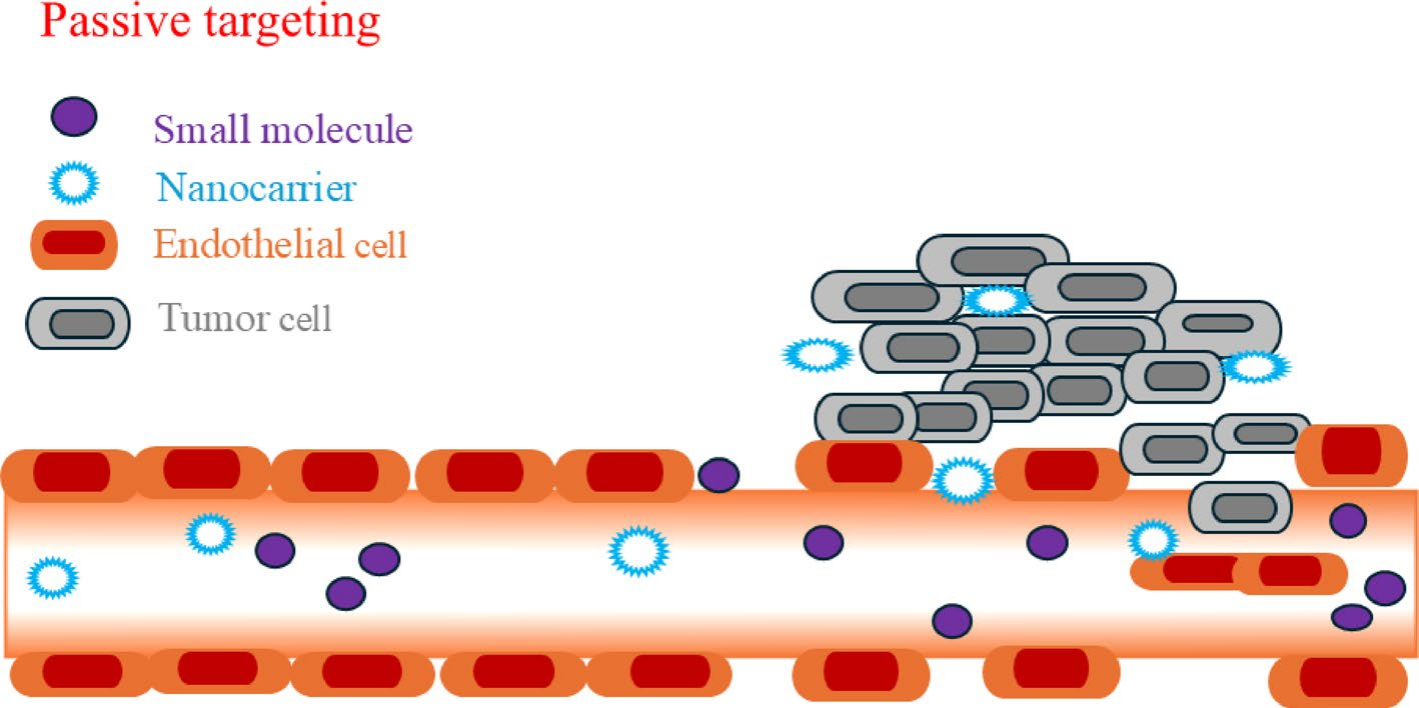
Passive targeting of nano carriers

Activated targeting moieties like antibodies and peptides are coupled with drug molecules, then attack the target site. In passive targeting, drug carrier is circulated in blood and reaches the target site through changing pH, temperature, size, shape, etc. Targeted drug delivery is changing their biological capability through active and passive processes. Through inactive targeting, therapeutic carriers could bind specific tissue cells. But for passive targeting, drug molecules reached the target cell. NPs are easily passed through the blood–brain barrier, because of the strong bond is observed by hyphaemia mannitol. Passive targeting systems are able to recover tumor tissue adopted in (Scheme 8). Nano systems are able to use the structural features of tumor tissue for passive targeting, whereby they have started the angiogenesis to overcome this problem.

**Scheme 9:**
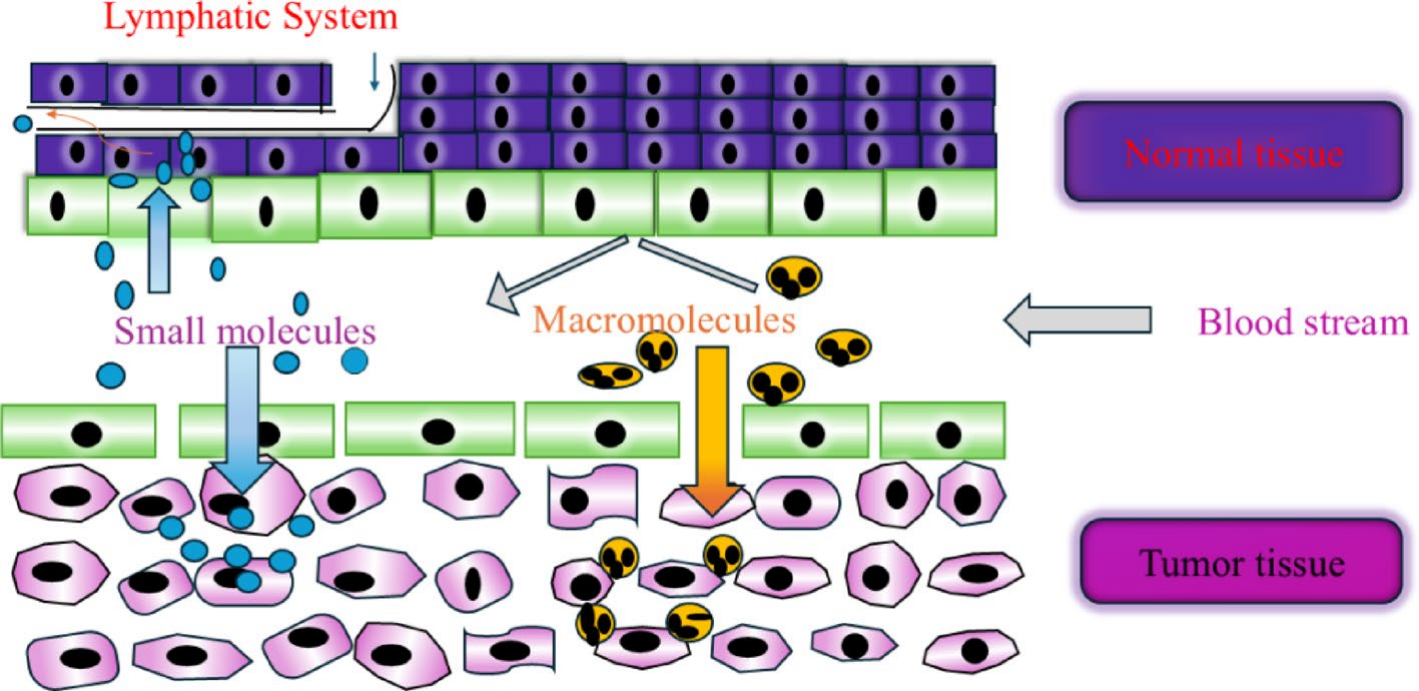
Passive targeting using tumor permeability capillary network

The size of the pores depends on the type of tumor. In addition, due to the lack of an efficient lymphatic system in tumor tissue, the interstitial pressure at centre of tumor is greater than the surrounding environment. Many drug delivery systems used to enhance permeation retention of tumor tissue. When the tumor size was 2 mm or more, it becomes permeable to start the angiogenesis. This is observed due to the development of absorption capability of waste food and oxygen. This phenomenon demonstrates abnormality in the basement membrane, lack of endothelial lining prostheses, and formation of leaking vessels. Different sizes of pores (100–1000 nm) are usable for passing the tumor cells. This feature has shown two important characteristics, such as (i) capillary endothelial malignant tissues are more unequal than healthy tissue due to their higher penetrability and (ii) absence of lymphatic drainage in the tumor area. As a result, by binding the chemotherapy drug with a suitable polymer, tumor permeability capillary network was observed by passive targeting (Scheme 9). These phenomena clearly indicated that a nano drug carrier was used to leave the vessel tumor to control endothelial tumor cells. The overgrowth and proliferation of cancer cells is most biological methods, which are unable to meet oxygen and energy due to the development of mildly acidic environment in the tumor environment. Through designing and manufacturing the drug-containing polymer NPs can change the shape in an acidic environment inside the tumors.

There are two problems happened for these methods, such as (i) drug being released before reaching the tumor and (ii) damaging the tissues around the tumor, because they showed a higher distance of acidic environment from the tumor. Therefore, the acidity of the tumor is not much lower than the natural environment of the body. The design of NPs does not release the drug in natural environment of body and its release capability is difficult. The enormous development techniques of drug efficacy could provide essential information about therapeutic efficacy for treatment of various diseases in health. Drug delivery mechanisms are successfully employed to improve human cells. However, these studies represent advanced challenges in developing successful delivery of drugs at target sites. Different nano-based drug delivery systems are currently deliberating advances in nanotechnology for designing the different varieties of nanomedicines. Thay are prevented various diseases using biocompatible NPs and nanorobot materials resulting in convenient administration routes, including lower toxicity, fewer side effects, improve biodistribution and extend drug release capability, respectively.

#### Cell-NPs interactions

**Scheme 10:**
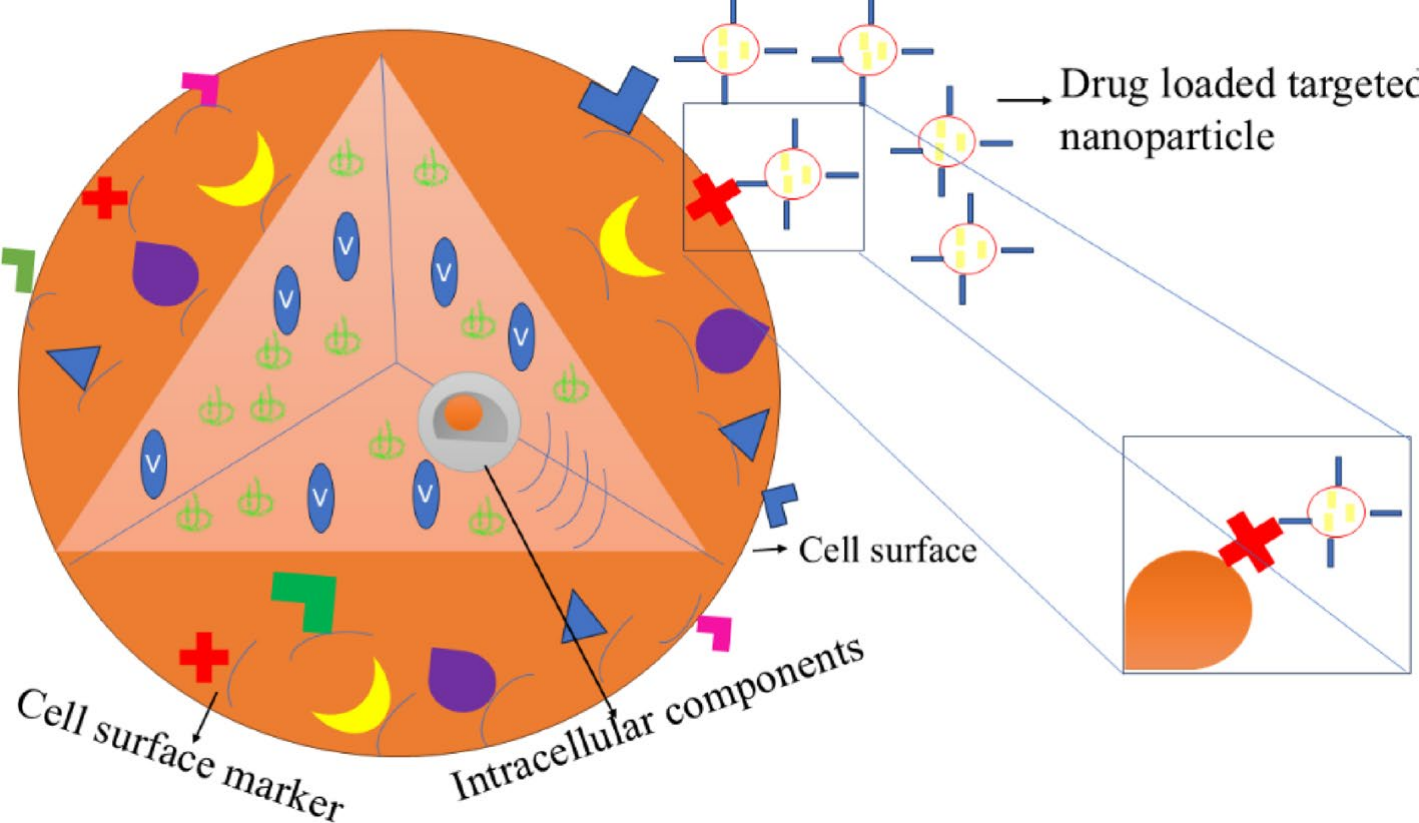
Cell-NPs interaction due to antibody antigen

NPs have been used to control different administration routes, such as oral, nasal, and intracellular cell surfaces. It also acts as an effective material to deliver drugs for developing various biomedical areas, such as cancer therapy and vaccine. NPs have been widely explored as biodegradable drug delivery devices due to their great rewards over conventional systems. Ideally, these drug delivery nanotechnologies are characterized by a single-dose treatment over a long time. The release of therapeutic compounds from NPs is settled by surface or bulk regions, chemical, biological degradation, diffusion through the pores, and release from the surface. NPs also need to ensure high transfection efficiencies for gene delivery. DNA or RNA is a genetic material used to ensure control gene expression through incorporating into the NPs, allowing them to protect genetic material from nuclease degradation. Similarly, genetic material is released from drugs in a controlled way during a certain time period. The genetic material is entrapped in the matrix and slowly released due to presence of diffusion and matrix degradation effects. The phagocytosis and pinocytosis pathways are chosen for internalization of nanocarriers. Here, research expressed the idea of clathrin-mediated endocytosis or caveolin-mediated endocytosis (Scheme 10).

The cellular uptake of NPs is subjected to change in surface charge, size, and shape, respectively. However, phagocytosis is related to larger sized particles, hence associated with macrophages, neutrophils, and monocytes to exhibit biological applications.

## PK/BD/clearance & toxicity

### Pharmacokinetics

Pharmacokinetic analogues is a mathematical technique, acting as surface-modified drug delivery systems to mimic cell surfaces [189, 190]. NPs are absorbed, distributed, metabolized, and excreted in the body, whereby they can enhance stability, prolong circulation, enable target-specific, and control drug delivery, respectively. These techniques are influenced by particle size, charge, surface modifications and pegylation, respectively. They are enabled to show passive tumor targeting by enhanced permeability and retention effect. At first, NPs are passed inside metabolism, liver, spleen, and lymphatic system by mononuclear phagocyte processes. This is very crucial predicting the complex biodistribution of NPs by incorporating anatomical parameters into tissue cells through changing the blood flow and physiological factors. For simulating the long-term multi-organ accumulation of NPs in the liver, spleen, and lungs can exhibit toxicity. NPs can be used for delivering drugs due to nonspecific distribution and unwanted toxicity, increased the extent of tissue-specific accumulation, patient compliance, and favorable clinical outcomes, respectively [191]. Furthermore, the nanocarrier systems can increase the drug bioavailability and sustain drug release in the target tissue. They are easily solubilized the drugs used for intravascular delivery and improved the stability of therapeutic agents against enzymatic degradation. For smart nanostructures, drug design is a fundamental topic to understand the NPs interaction with biological systems. The particle size, surface charge, surface modification using targeting ligand, pegylation functionalization, and composition can influence the pharmacokinetics of NPs. Particles with specific characteristics could exhibit long blood circulation time in bloodstream due to delayed release. Those effects enhance the cellular uptake and organ accumulation capability [192]. The pharmacogenomics allows for the identification of genetic variations to influence the individual drug responses for facilitating personalized treatment strategies. The pharmacology complements are utilized computational model biology systems to identify potential biomarkers and optimize drug efficacy. These concepts underscore importance of pharmacokinetic analogues in advancing drug development.

### Toxicity and safety concerns

NPs possess significant toxicity risks due to different sizes and high surface-area-to-volume ratio, whereby they can easily pass inside the tissues and biological barriers. The natural source of toxicity is observed due to presence of heavy metals, engineer materials, and fibrous shapes of carbon nanotubes. Different key safety concerns including respiratory, cardiovascular, neurological damage, oxidative stress, inflammation, cellular injury, inflammation, and apoptosis cell death are influenced by the primary drug delivery and release mechanism observer due to generating of ROS. NPs accumulate on the upper surfaces of affected organs, such as lungs, liver, and brain, necrosis can control the therapeutic efficacy through the metabolic processes. The safety concerns and inhalation in animal models are most dangerous routes particularly damaging the lung cells, whereby studies on drug loading NPs have shown interesting aggression and inactivity natures. The carbon-based nanotubes are exhibiting bio persistent capability in a body and cause long-term harmful effects. The toxicity studies are necessary for developing safer biocompatible NPs due to adverse effects, including respiratory, cardiovascular, neurological, and cancer diseases-controlled effects [193].

The toxicological mechanism of NPs is still not fully understood. However, several studies have shown that the toxicity of NPs is mainly reduced due to the generation of ROS and the induction of oxidative stress. The ROS are highly reactive molecules that can damage cellular components, proteins, lipids, and DNA, leading to cell death. NPs can also induce inflammation by activating the immune system through releasing the inflammatory cytokines. The toxic effect of NPs on human health has attracted more attention, and its toxicological mechanism is complex and multifactorial. Further research is needed to understand mechanism and develop effective strategies to mitigate adverse effects. Therefore, development of safe and biocompatible NPs is crucial in various research fields, such as medicine, electronics, and energy, respectively.

## Translational & regulatory considerations

### Regulatory challenges

Regulatory challenges of NPs are unique, size-dependent properties, which often render traditional risk assessment frameworks inadequately. Different regulatory challenges are key issues, due to its lack of standardized, specific regulations, hindering approval and safety assessments. Major obstacles are involved due to its inconsistent definitions, difficulties in toxicity testing, unpredictable environmental and biological behaviour, and ethical concerns [194]. Using inadequate toxicological test, it may not accurately assess the unique hazards passed the high reactivity NPs. The safety and toxicity studies confirmed that NPs can be used for DNA damage, inflammation, and oxidative stress. The long-term impact of nanomaterials on human health and environment is not well understood leading to difficulties in establishing exposure limits. Regulatory bodies are identifying enabled nano products to ensure transparency. Balancing nanotechnology is needed for transparent safety data during approval processes. The drug delivery systems follow different conventional rules whereby different challenges are observed for safety concerns. The environmental contamination showed the unknown effects of NPs; those are potentially high risks to handling the engineered nanomaterials. Regulatory challenges and limitations discussions are associated with novel DDS including development of complex preparation, regulatory considerations, engineering scalability, stability and potential effects. The significant challenges are ensured the successful translation from laboratory to clinical practices. The novel DDSs are not only shown the versatile applications across various therapeutic areas on improving treatment outcomes, but also control the harmful diseases, such as cancer, cardiovascular diseases, neurological disorders, and infectious diseases, respectively.

### Central hypothesis

The central hypothesis is a unique parameter. The size-dependent physicochemical properties specifically showed high surface area to volume ratio enables them to enhance biological, chemical, physical targeted drug delivery properties and increased reactivity through compared the bulk counterparts [195]. These engineering characteristics allow them to cross biological barriers, such as blood–brain barrier, and interact with cellular components. The fundamental properties are changed drastically from shrink like materials to nanoscale, exhibiting unexpected optical, magnetic, and electronic behaviours. A major hypothesis of NPs is that exposure through inhalation, which can trigger adverse effects such as oxidative stress, inflammation, and cellular damage in the brain. NPs are hypothesized to improve therapeutic outcomes by enhancing specific drug delivery into tumor cells. By controlling the size, shape, and surface properties, NPs could exhibit transformative applications in medicine, technology, and industry, respectively. The overall hypothesis is that drug-loaded NPs exhibit deleterious effects on brain health through activation of microglia, triggering low-grade chronic neuroinflammation and subsequent regulation of brain function. The main aim is to test the low-dose chronic exposure, rather than acute apoptotic response to higher NPs at concentration (> 0.5 g/mL). On the other hand, higher doses may not reflect the ambient environmental exposure in the real-world. Here, we have discussed an in vitro model by exposing microglia to low concentrations of AgNPs (20 nm) by profiling immune response, specifically release of neurotoxic pro-inflammatory cytokines in presence of upstream regulator immune responses [196].

### Limitations

A holistic and forward-looking study of NPs showed various limitations showed ground breaking potential in medicine, electronic, environmental remediation, etc. Its clinical and commercial adoption is currently hindered by challenges for manufacture, and regulation steps. Many NPs lead to long-term potential health risks and accumulated in liver, spleen, and kidney to generate ROS, resulting in DNA damage and cell death. NPs are deeply penetrated into tumors and rapidly cleared the immune system before reaching the target site. The monodisperse NPs are technically challenging and expensive on a large-scale, high-quality production. There is limited understanding of long-term ecological consequences of NPs release into environment, particularly persistence in natural ecosystems. That can be used for forward-looking perspectives and future directions. To overcome toxic synthetic methods, future DDS s shifting towards green synthesis using plant extract, bacteria, and fungi to create eco-friendly, biocompatible NPs. The development of stimulus-responsive tumor is crucial for increasing efficacy and reducing target side effects. There is an urgent need for explicit regulatory frameworks for the handling and disposing of nanomaterials. In summary, we analyzed nano research through moving away from smaller NPs toward sophisticated design and environmentally sustainable nanostructures. NPs can accumulate in healthy organs and tissues, leading to potential toxicity and long-term side effects. Additionally, it is limited to the long-term effects of metallic NPs on the human body, raising concerns about their safety and efficacy in clinical applications. Despite their potential in cancer treatment, they showed efficacy in clinical applications. It is crucial to conduct further research and rigorous testing to fully understand the risks and benefits associating with metallic NPs. Additionally, regulatory agencies should establish guidelines and protocols to ensure the safe and responsible of NPs when used for in medicinal fields. Henceforth, long-term studies are needed to assess the potential side effects and interaction of metallic NPs with other medication treatments. Additionally, it is important to involve multidisciplinary clinicians and regulatory experts to evaluate ethical implications and address any potential societal concerns for cancer treatment.

### Applications

**Scheme 11:**
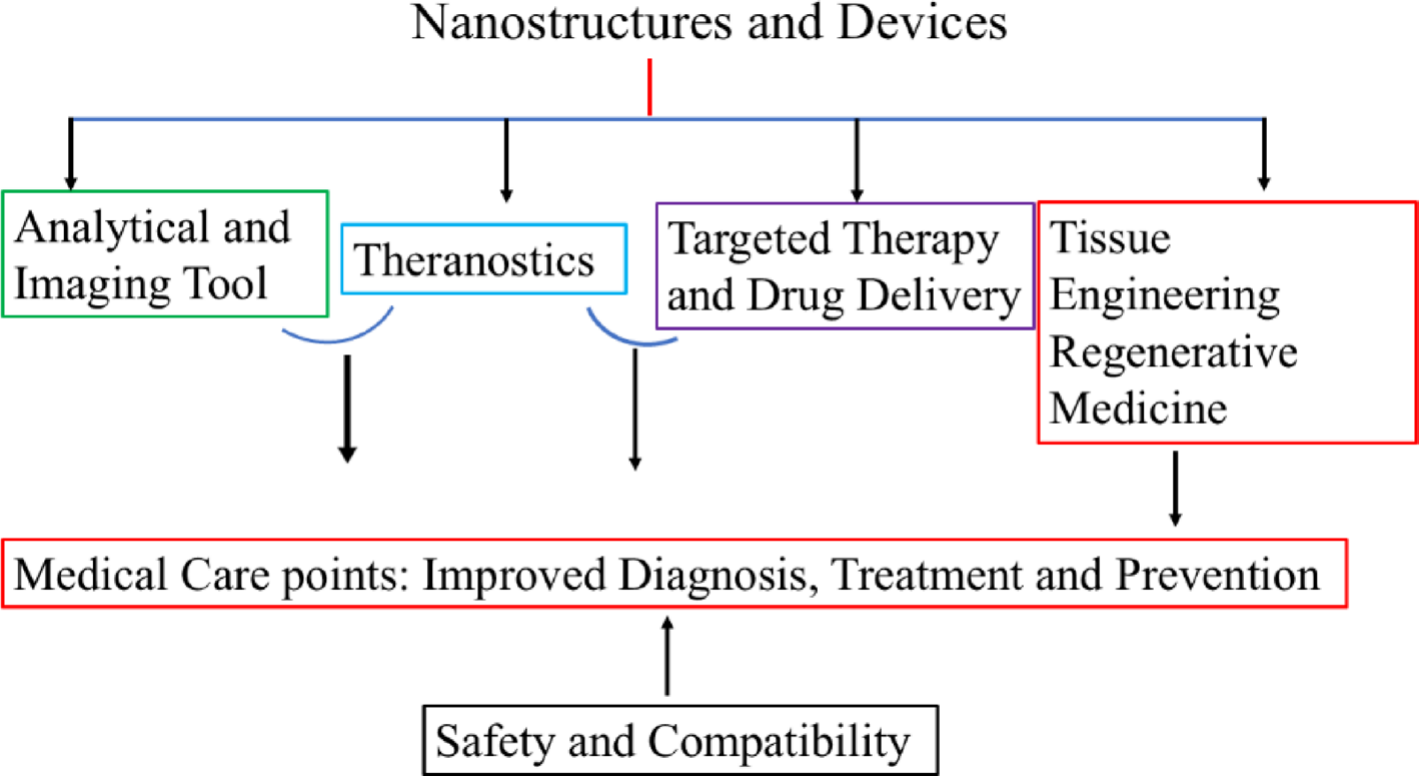
Application of nanomedicine in different biomedical research fields

NPs are exhibited various applications such as diagnosis, drug delivery, sensory, and actuation in living organisms (Scheme 11).

Nanotechnology could provide interesting knowledges for development the material science depending on sizes. NPs showed different applications such as medical, commercial, ecological sectors, electrical, mechanical, optical, microelectronics, aerospace, pharmaceutical industries and imaging properties, respectively [197]. They can increase the concentration of NPs in groundwater and soil, resulting in assessed environmental risks. Natural NPs play an important role in the solid-water partitioning of contaminants due to their high surface to mass ratio. NPs have been identified as printed electronics through interacting with traditional silicon. NPs are used for energy harvesting purposes due to their large surface area and optical and catalytic nature. They have shown photocatalyst energy and electrochemical water splitting for generating the energy. Recently, nanogenerators convert mechanical energy into electricity using piezoelectricity to generate the energy [198, 199].

The In vivo epidemiological studies describe a growing incidence of viral infections that can improve worldwide. Different viruses affect human cells and create negative health and socio-economic growth. To remove this disease problem, different nanomedicine techniques, such as antigen shifting, drafting, non-specific drug targeting, and suboptimal drug concentrations are essential for unproductive remedial treatment against viral infections [200]. Nanotechnology-based drug delivery is an alternative approach used to develop therapeutic efficiency through altering the physicochemical properties. Non-specific cell targeting and suboptimal drug concentration are most vital issues to improve drug resistance against novel coronavirus. Nanotechnology-based programmable drug delivery is an alternative approach used to satisfy optimal drug concentration at the target site. That can reduce dose frequency, bioavailability, drug degradation; inhibit cellular system and improve cell-target capability, respectively. Nano-dimensional configuration with improved permeability and retention effects can increase surface area to volume ratio, which is a confidential aspect making a promising drug delivery system. Li et al. [201] reported that synthesized zidovudine NPs exhibit drug delivery activity. A nanocarrier is another type of drugs delivery vehicle that can deliver drug into a host body whose size is < 500 nm. This advanced technology shows promising results to improve the quality of drugs by up-regulating solubility, stability, bioactivity, and regulating toxicity in host cells. NPs have interesting properties in different branches of medicine due to its drug delivery capability and therapeutic efficiency [135]. Surface-active polyethylene oxide and polylactic acid used as intravenous administrative drugs used to enhance the interaction ability off antigen–antibody in tissue cells [19].

**Scheme 12:**
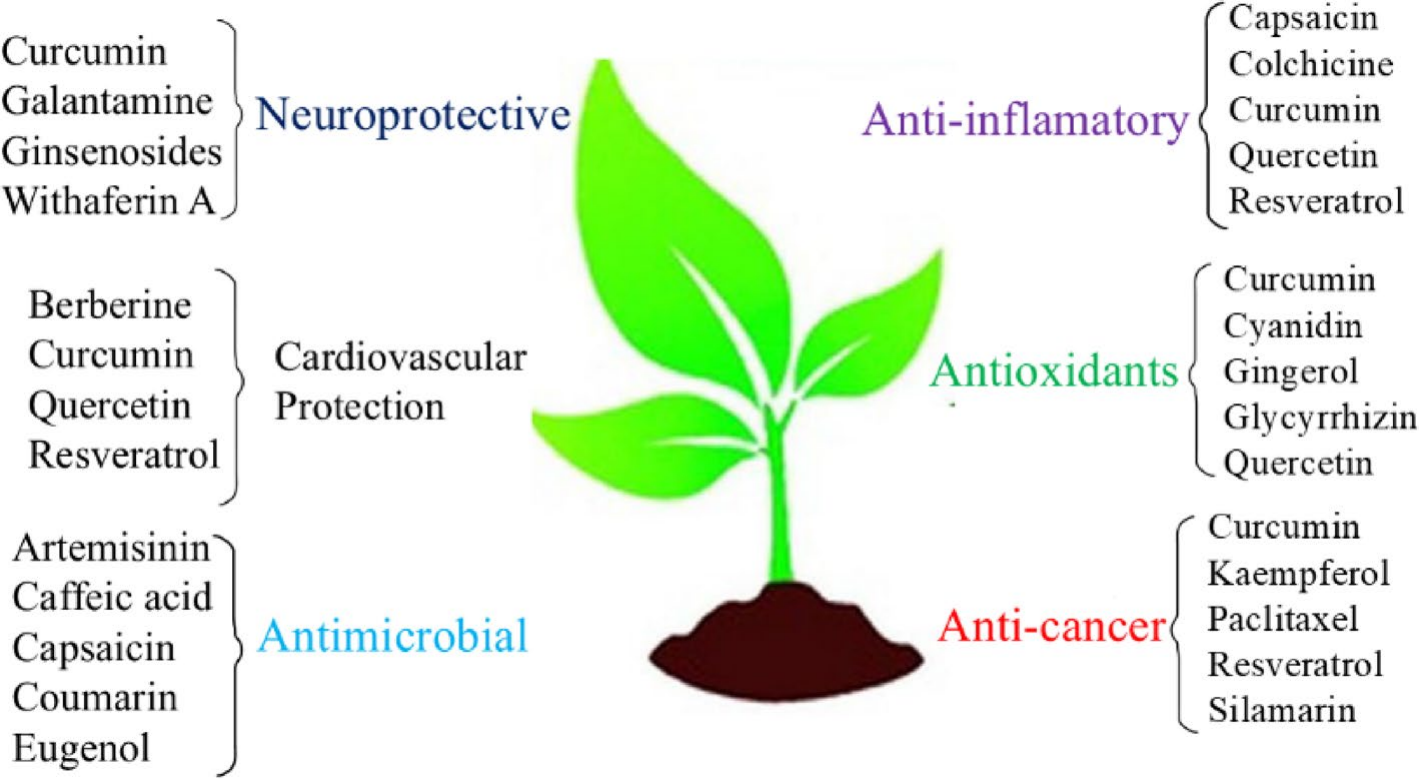
Different extract was obtained from natural compounds used in nanomedicine

Plant-based natural products used as medicine in human cell lines against various viral and bacterial diseases [202]. The opportunities, challenges, and clinical applications of nanomedicine are briefly discussed through natural synthesis processes. Natural compounds used for disease purposes due to the presence of various potential activities. Nanoscale-sized materials are used in emerging area of nanoscience, nanotechnology, solar energy renovation, catalysis, medicine, and water treatment, respectively. Their potential demand may have conveyed by green synthesis method. According to global effort, green chemistry and chemical processes are interacting topics to reduce generated hazardous waste materials from tissue cells (Scheme 12).

Using the safe chemical products and controlling the maximum efficiency of chemical processes, implementation of sustainable processes should be adopted for minimizing the fundamental aspect. Their different molecular backgrounds helped to discover drugs [203, 204]. However, natural compounds are biocompatible and low toxic, which can represent greater challenge in medicine. Despite several advantages, different pharmaceutical companies have been produced natural product-based drugs and explored their bioavailability for biological activity purposes. The natural compounds are used to control different important diseases, such as cancer, diabetes, cardiovascular, inflammatory, and microbial diseases, respectively [205–207]. Large-size NPs possess major challenge for drug delivery because they are unstable, lower bioavailability, deprived solubility, less absorption in skin surfaces, specific target drug delivery, and tonic efficiency, respectively. Therefore, researcher used small size NPs, those are attacking on specific targeted area of body parts and solved critical diseases with immense success. Nanotechnologies are offering lot of opportunities to develop pharmaceutical research. Some metal NPs have broad spectrum, preventing virus growth in human body. Franchi et al. [208] reported AgNPs are mainly used as antiviral agent for preventing the growth of microorganism. Surface of NPs are modified through nanoscale biocides using quaternary ammonium and phosphonium salts, through controlling the oxidation of microbial membranes. Hence, nanotechnology plays a significant role in advanced medicine and drug formulation.

## Research gaps

Based on recent research through studying different literature, we found that the field of nanotechnology has been shown several critical research gaps, primarily focusing on long-term environmental safety, standardized toxicity testing, and transforming nanomaterials in real-world scenarios. The long-term, low-concentration effects of nanomaterials, especially carbon-based in natural ecosystems are largely unknown. There is a lack of data on whether biological systems can adapt to NPs showing reversible toxicity. The reproductive toxicity and long-term intergenerational impacts of NPs exposure in infancy, suggesting potential for adverse effects on sperm and placental transfer. The influence of environmental corona, metals and polymers are not well-understood for toxicity. Most studies focused on cellular toxicity assessed the risks at the community and ecological levels. There is an urgent need for globally standardized nomenclature, characterization, and reference materials, particularly for nano plastics and complex composites. Current technologies are inadequate for counting low concentrations of carbon-based nanomaterials in environmental matrices. There is a research gap between laboratory-scale synthesis and mass production that maintains the required quality, uniformity, and stability of NPs. The NP-based treatments have minimal difficulties in predicting long-term in vivo biodistribution and overcoming immune system clearance. More in-silico computational model is needed to predict the biological behavior of novel nanoforms without relying solely on time-consuming in vivo tests. Research has confined to the diverse shapes and compositions of environmental NPs for detection, identification and quantification remain technologically challenging in the environment.

## Future directions

The future research direction of nanotechnology is transformative. It can be used as a promising advancement material in medicine targeted drug delivery and diagnostics. On the other hand, NPs exhibit different electrical activity due to the presence of unpair electrons used for electronics chips, flexible displays, quantum computing energy materials, and lighter composites focusing on safety and ethical development. In recent years, for higher development of nanotechnology, NPs have been used for real-time diagnostics and customized treatments for detecting diseases at much earlier stages. Development of nanoscale transistors and ultra-dense memory control smaller and faster device is more appropriate for cancer cell and tumor cell treatments. NPs are more efficiency for the solar cell battery, aerospace, construction, and automotive industries due to the presence of different energetic capabilities. Nanofiltration techniques are more suitable for removing the contaminants and desalination of nano catalysts through reducing emissions and cleaning agents. Self-healing, anti-scratch, and antimicrobial effects are the most prominent techniques in modern fundamental research. Integrating safety assessments into NP development by creating smart, responsive materials and systems is the most vital topic in future research. Using an eco-friendly reducing agent, we have developed biocompatible NPs through high screening technologies that can help to understand the toxicological mechanisms. The toxic behavior of NPs in biological systems can be ensured the safety concern in the future perspectives. It is important to anticipate future trends and challenges to guide further research and development efforts in nanotechnology, biomaterials, and personalized medicine field.

## Conclusion

Nowadays, recent advances in nanomedicines as well as novel diagnostic methods using drugs in the target cells are hot topics in nanotechnology fields. Based on this theme, different nano-dimensional materials, including nanorobots and nano sensors are applicable for diagnostic treatment. Exactly delivering the drug molecules to specific target sites and imaging activities of bioactive materials have been shown interesting biological behaviours. Different synthesis and physicochemical properties of nano-based drug molecules could provide the knowledge about solubility, absorption, bioavailability, and control release, respectively. The novel natural biomaterials have been shown biodegradability, biocompatibility, renewability, and lower toxicity, respectively. The efficacy of these natural products has greatly improved their nanotechnology research using gold, silver, cadmium sulphide, and titanium dioxide polymeric NPs. Similarly, different SLNPs, such as liposomes, micelles, and dendrimers are chosen as capping agents, those are bound with nanocarriers and passed through the body cells then started their activity. To shed on light, the main focus of this review is development of nanomedicine, drug therapy, and diagnosis for controlling breast cancer and tumor-like death diseases. Nanomedicine has opened up the newer avenue by discover the administration routes in biological systems. NPs are situated in white blood cells searching for disease signals or cleaning blood vessels. NPs can be engineered to cross the blood–brain barrier or release medication only at tumor sites by reducing systemic side effects. They have been used for dissociation of pollutants like heavy metals with higher precision than conventional filters. It is used for green nanotechnology using neem and ginger plant extracts rather than hazardous chemicals. The novelty in nanotechnology field lies in understanding how the size, shape, and surface chemistry of nanoparticles influence their interaction with biological systems. The higher percentage of atoms are located on the NPs surface, drastically increasing reactivity and altering fundamental physical behaviours. As particle size decreases, the surface area increases exponentially relative to volume, making them highly efficient for catalysis and adsorption. In the nano-range quantum physics dominate tuneable optical properties, whereby AuNPs are appearing red or purple rather than yellow. Many NPs exhibit enhanced electrical conductivity or superparamagnetic properties due to the presence of powerful magnetic external field. Using different drug-releasing mechanism, this review could exhibit nontoxicity and biocompatibility for further development of biological sites. SiNPs are known for variety of applications, including wound dressings, water purification antimicrobial activity, environmental impact, low toxicity, sol–gel processing, hydrothermal synthesis, and laser ablation, respectively. The toxicity of NPs is a critical area of research due to their potential impact on human health and environment. This knowledge is essential for developing safe NPs for various applications and mitigating potential risks. The development of NPs can target specific biological processes, leading to new diagnostic and therapeutic strategies, DDS, biosensors, tissue engineering scaffolds, etc*.* CNPs are exhibited exceptional mechanical, thermal, and electrical properties, making them suitable for materials, electronics, energy storage, sensors, and biomedical devices, respectively. Graphene NPs are extremely thin, strong, and conductive, making them ideal for applications in electronics, energy storage, and composite materials.

## Data Availability

No datasets were generated or analysed during the current study.

## Declarations

### Clinical trial number

This review article does not involve any in vivo animal experimental model, so clinical trial number is no needed.
